# Dietary Fibre in Pea Cotyledon Used as a Protein Source Provides Multidimensional Protection Against Colonic Protein Putrefaction

**DOI:** 10.3390/nu18203347

**Published:** 2026-10-13

**Authors:** Suman Sunita Mishra, Chunlong Mu, Caroline Kim, Carel Jobsis, Duncan Hedderley, Anika Hoogeveen, Christine Butts, Sheridan Martell, Hannah Dinnan, Suzanne Middlemiss-Kraak, Halina Stoklosinski, John Monro

**Affiliations:** 1 Bioeconomy Science Institute, Private Bag 11600, Palmerston North 4442, New Zealand; suman.mishra@plantandfood.co.nz (S.S.M.); caroline.kim@plantandfood.co.nz (C.K.); carel.jobsis@plantandfood.co.nz (C.J.); duncan.hedderley@plantandfood.co.nz (D.H.); anikahoogeveen@hotmail.com (A.H.); chrissie.butts@plantandfood.co.nz (C.B.); sheridan.martell@plantandfood.co.nz (S.M.); hannah.dinnan@plantandfood.co.nz (H.D.); suzanne.middlemiss-kraak@plantandfood.co.nz (S.M.-K.); halina.stoklosinski@plantandfood.co.nz (H.S.); 2 Cumming School of Medicine, University of Calgary, 2500 University Dr NW, Calgary, AB T2N 1N4, Canada; chunlong.mu1@ucalgary.ca; 3 Riddet Institute, University Avenue, Fitzherbert, Palmerston North 4474, New Zealand

**Keywords:** colorectal cancer, meat, pea cotyledon, faecal bulk, fermentation

## Abstract

**Background/Objectives:** Substitution of plant-based for animal-based protein sources has been recommended to reduce global non-communicable disease burdens, including colorectal cancer. Objective: The goal of this study was to determine possible ways that pea cotyledon may modulate hindgut concentrations of protein putrefaction products implicated in colorectal cancer by comparing diets based on casein, meat or pea cotyledon in a rat model. **Method:** Rats (*n* = 8 per treatment) were fed for 9 days on 18.6% protein diets containing casein, meat, or yellow split peas as sole protein sources. Faecal samples were collected daily from day 6 onwards and gut samples post-mortem for analysis. Protection by hindgut contents against protein putrefaction products was assessed using a novel secondary in vitro fermentation of the rat faeces with human faecal inoculum and added protein. **Results:** Compared with the average of non-pea diets, which was similar to one another, pea cotyledon increased caecal digesta content (101%, *p* < 0.001), faecal dry matter (116%, *p* < 0.001), faecal water-holding capacity (66%, *p* < 0.001) and hydrated faecal mass (235%, *p* < 0.001). Caecal putrefaction product concentrations (*p*-cresol, indole, and phenol) were 72% lower on the pea diet compared with the non-pea diet average (*p* < 0.001). The pea diet altered the microbiota composition and downregulated tryptophanase activity while increasing butyrate production by 236% (*p* < 0.001). During ex vivo fermentation with added protein pea-fed rat faeces suppressed protein putrefaction and promoted butyrate production, whereas faeces from other diets were as ineffective as the no-faeces control, showing that distal transfer of more than enough pea polysaccharides to sustain distal colonic saccharolytic fermentation had occurred. **Conclusions:** Dietary fibre associated with protein in pea cotyledon protects against protein putrefaction products by bulk dilution, a decreased residence time, changing microbiome composition and function, and distal displacement of fermentable dietary fibre, therefore providing multidimensional protection.

## 1. Introduction

The EAT-Lancet Commission has recommended a move from animal-based to plant-based sources of dietary protein for both human and planetary health. Improvements in public health are predicted to follow with a reduced incidence of major non-communicable diseases [1].

Colorectal cancer (CRC) is a non-communicable disease of elevated incidence in populations with a high meat and low dietary fibre intake [2,3,4,5], in which a contributing risk factor is a relatively high colonic protein concentration combined with depleted carbohydrates, leading to the generation of carcinogens by protein fermentation (putrefaction). The risk of CRC could be reduced by substituting legume cotyledon for animal meat as a source of dietary protein because of the direct and indirect effects of plant cell walls (dietary fibre) associated with protein in pea cotyledons.

The legume fibre could act in several ways. It could increase hydrated colonic bulk with the dual effects of increasing the transit rate (reducing the colonic residence time) [6] and reducing chemical activity by dilution. It may also provide fermentable polysaccharides to the distal colon, sparing colonic protein from the putrefaction that produces carcinogenic by-products [7] while supporting the development of a beneficial microbiome [8] and the production of protective short-chain fatty acids, such as butyrate [9].

On the other hand, the encapsulation of protein by robust cotyledon cell walls in legumes may reduce protein digestion in the foregut and increase colonic protein loading [10]. This could, in theory, counteract the protective effect of legume dietary fibre against protein putrefaction. Therefore, there are counterpoised factors that may determine the net advantage of legume protein sources over meat protein in protecting against CRC.

One way to show the role of factors determining the net impact of plant versus meat protein sources on protein putrefaction is to use a valid animal model. The rat has proven to be a useful model in studies of the faecal bulking effects of foods [11]. Like humans, the rat is a monogastric hindgut fermenter with a diverse bacterial flora that has a fermentative capability like that of humans [12,13]. In both rats and humans, faecal properties depend on the nature of unfermented food and endogenous residues in the faeces and the associated microbial biomass, which together determine the water-holding capacity of the faecal mass. Although faeces of rats are released as partially dehydrated pellets in vivo, when allowed to passively imbibe water they reach the same water content as human faeces, making them an accurate model for analysis of effects of foods on the human faecal mass [14].

The rat monogastric model for assessing the faecal bulking capacity of foods may also be used to measure fermentable dietary fibre transfer to the distal colon, if one assumes that with an ample fibre loading there will be some spill-over of fermentable fibre into the faeces. A secondary in vitro fermentation of the faeces should then reveal the extent to which more than enough fermentable fibre has reached the colon to maintain distal colonic fermentation. And if protein is added to the secondary fermentation, it may reveal the capacity of the fermentable faecal fibre to protect against the production of harmful protein putrefaction products in the colon.

Changes in microbiota composition and activity in response to a high protein–low carbohydrate colonic substrate is also likely to be a critical component of the switch from polysaccharide fermentation to protein putrefaction and may be examined concurrently with analysis of faecal bulking. Faecal bulking analysis of a food is usually run over a 4-day balance period after a lead-in of several days, 5 days in the present study. Although such a short turn-around may not give the hindgut microbiota time to completely adjust to a change in diet [15], the bulk of adaptation to a change in colonic substrate has been shown to be rapid, occurring within a few days [15,16]. Therefore, microbiota changes in rats fed foods as different as legume cotyledon and meat should appear by the end of a nine-day faecal bulking trial and help to illuminate the relative effects of the two foods.

This paper reports results of a study in which rats were fed diets containing similar concentrations of protein in the form of pea cotyledon, casein, and meat (beef) for 9 days, with the last 4 days being a balance period during which samples were collected. Outcomes included effects on the hindgut concentration of protein putrefaction products, changes in the hindgut microbiota, changes in faecal bulk and hydration. In a novel in vitro assay of the distal extension of the protective effect of residual fermentable polysaccharides against protein putrefaction, the ability of the rat faeces to suppress putrefaction in the presence of added protein and human faecal inoculum was measured.

We hypothesised that equi-protein substitution of legume (pea) for meat would decrease hindgut concentrations of protein putrefaction products and favour the development of a healthier microbiota attuned to polysaccharide fermentation rather than protein putrefaction. And because of a greater hydrated faecal mass resulting from the water-holding capacity of pea cotyledon cell walls, protein putrefaction products would be diluted, and distal colonic transfer of fermentable fibre would be extended, further protecting against protein putrefaction. The protection given against the production of protein putrefaction products by distally transferred fermentable carbohydrates would be shown by the ability of faecal mass from the pea-fed rats, but not from meat-fed rats, to suppress putrefaction of added protein during a secondary in vitro faecal fermentation with human faecal inoculum.

## 2. Methods

### 2.1. Meat and Pea Preparations

Lean minced beef steak (5% fat) was purchased from “The Mad Butcher” in Palmerston North, New Zealand. It was cooked by stirring over boiling water in a double boiler until all sign of redness had disappeared, then frozen and freeze-dried. The dried meat was then pulverised to a fine powder in a Wareing blender and passed through a 1.0 mm sieve. A subsample was weighed, extracted with acetone, filtered, dried and reweighed to estimate fat and protein content by difference. The proximate composition was 63.35% water, 6.5% fat, and 30.2% protein. The corresponding New Zealand Food Composition Database values for braised beef steak (Record M1178) are 60.7 water, 9.2% fat and 30.2% protein (www.foodcomposition.co.nz/downloads/concise-14-edition.pdf (accessed on 3 February 2024)), so a value of 30.2% protein was assumed.

The yellow split peas were McKenzie’s Australian Yellow Split Peas (Woolworths, Palmerston North, New Zealand). They were rinsed, soaked for 2 h and cooked intact by boiling for 10 min, frozen and freeze-dried. They were briefly pulverised in a Waring blender and passed through a 750 µM sieve, and any retained particles re-ground and again sieved until no material was retained. The protein content was based on the values given in the nutrient information panel (23.3%).

The dietary fibre value given in the nutrient information panel was 25.4%. Dietary fibre, determined in triplicate as the 80% ethanol-insoluble residue after peptic and pancreatic digestion of the cooked, dried, and powdered split peas used in the present study, was 21 ± 5%, and this value was used in calculations of faecal survival of pea dietary fibre.

### 2.2. Diets

The animals were fed casein (Diet 1), meat (Diet 3) and pea (Diet 4) diets all at 18.6% protein content and one casein diet (Diet 2) at twice the level (37.2%) of protein (Table 1) to check the influence of protein intake, although the main hypothesis was tested with diets 1, 3 and 4. The diets were formulated to all contain the same animal fat content by partial beef fat substitution of starch in the diets not containing meat. All vitamins and minerals used were of reagent quality. All diets had a powder consistency after mixing, but the rats had free access to water and appeared to have no difficulty ingesting them (Supplementary Figure S1).

### 2.3. Animals and Sampling

This study, including protocol and all aspects of animal welfare, was approved by the AgResearch Animal Ethics Committee (Palmerston North, New Zealand; Application no. 2328), according to the Animal Welfare Act 1999, New Zealand. Male Sprague–Dawley rats were born on site and raised from weaning on standard rat pellets (Diet 86, Sharpes Farm Feeds, Carterton, New Zealand) until the start of the trial when their weight was 268.6 ± 12 g (mean ± SD). Animals were included if assessed as healthy based on growth and appearance at the start of the trial.

The trial lasted 9 days, of which the first 5 days were a lead-in period, and the last 4 days were a balance period during which food intake, spillage and refusals were accurately recorded, and the total faecal output was collected from each rat. Power was calculated for a one-way ANOVA with four treatment groups using the ASAMPLESIZE procedure in Genstat (version 22, VSNI Ltd., Hemel Hempstead, UK 2023) and data in Table 5 of Paturi et al., 2012 [17]. The rats (*n* = 8 per treatment) were housed in hanging cages in a temperature- (23 ± 1 °C) and humidity (60 ± 5%)-controlled room at the Plant and Food Research small animal facility and fed 25 g/day of the diets. The rats were assigned without preference to the cages before the cages were linked to treatments and cared for by technicians blind to the hypothesised treatment effects. The rats were housed in pairs for the first 5 days, as directed by the animal ethics committee and housed individually for the final 4-day balance period to enable collection of samples from individual rats. To avoid positioning effects during the balance period the cages were arranged in a single 60-position frame as 8 consecutive linear blocks, each block containing one of each of the treatments positioned randomly within the block. The personnel responsible for rat feeding and faecal collection were blind to the diet composition and hypothesised effects.

The rats ingested most (80 ± 7%) of their diet allocation during the four-day balance period (Supplementary Figure S1), assessed as the difference between the sum of spillage and refusal and the 100 g feed allocation over the balance period. They were weighed at trial start, after 2 days and at days 5, 7 and 9, being the start, middle and finish of the balance period, respectively. The growth of all groups was uniform over the 9-day treatment period (Supplementary Figure S2).

At the completion of the trial the rats were euthanised by carbon dioxide anaesthesia and the contents of the caecum and of the anterior (proximal) and posterior (distal) halves of the colon immediately collected and frozen at −80 °C.

### 2.4. Analysis of Fermentation and Putrefaction Products

#### 2.4.1. Short-Chain Fatty Acids

The organic acids in caecum samples were quantified as previously described [18] with a slight modification. Frozen caecal supernatants were weighed and diluted 10-fold with 0.01 M of phosphate-buffered saline (PBS) containing 2-ethylbutyric acid (5.56 mM) as an internal standard. The final concentration of the internal standard was 5 mM. The PBS mixture was centrifuged at 3000× *g* for 5 min at room temperature, and 500 µL of supernatant was transferred into a fresh tube. Following acidification with 250 µL of concentrated hydrochloric acid, 1000 µL of diethyl ether was added. The organic acids in fermenta samples were quantified as previously described [19]. Fermenta samples were centrifuged at 3000× *g* for 10 min, and 100 µL of supernatant was stored at −80 °C. While still frozen, 400 µL of 0.01 M of PBS containing 2-ethylbutyric acid (6.25 mM) was added to the 100 µL frozen sample to make a 500 µL sample containing 5 mM of 2-ethylbutyric acid. After acidification with 250 µL concentrated hydrochloric acid, 1000 µL of diethyl ether was added.

Following vortex mixing and partitioning of the acids to the diethyl ether phase, the caecal and fermenta samples were centrifuged at 10,000× *g* for 5 min (4 °C). In a capped GC vial, 100 µL of the diethyl ether phase was derivatised with 20 µL of N-tert-butyldimethylsilyl-N-methyltrifluoroacetamide with 1% tert-butyldimethylchlorosilane (MTBSTFA + TBDMSCI, 99:1; Sigma-Aldrich, St. Louis, MO, USA) in a water bath at 80 °C for 20 min. To allow for complete derivatisation of lactate and succinate, the samples were left for 48 h at room temperature before analysis. Standards containing 2-ethylbutyric acid (5 mM) as an internal standard were prepared for derivatisation alongside the samples.

Analysis was performed on a Shimadzu capillary gas chromatograph system (GC-2010 Plus, Tokyo, Japan) equipped with an FID and fitted with a Restek column (SH-Rtx-1, 30 m × 0.25 mm ID × 0.25 µm) (Shimadzu, Canby, OR, USA). The carrier gas was helium, with a total flow rate of 21.2 mL/min and pressure of 131.2 kPa. The make-up gas was nitrogen. The temperature programme began at 70 °C, increasing to 115 °C at 6 °C/min, with a final increase to 300 °C at 60 °C/min, holding for 3 min. Flow control mode was set to linear velocity (37.5 cm/s). The injector temperature was 260 °C, and the detector temperature was 310 °C. Samples were injected (1 µL) with a split injection (split ratio, 10:1). The GC instrument was controlled and data processed using the Shimadzu GC Work Station LabSolutions software (version 5.132). Acquired data are expressed as µmol organic acid/mL fermenta or µmol organic acid/g caecal digesta.

#### 2.4.2. Protein Putrefaction Products

Ammonia: The samples were taken out at −80 °C and thawed at room temperature. Approximately 100 mg of the caecal content was accurately weighed into a 2 mL Eppendorf tube, 500 μL of distilled water added, and the tube contents made into a slurry by thorough vortexing, then centrifuged at 2500× *g* for 20 min. A 50 µL aliquot was taken and made to 500 µL with distilled water, then 400 μL of the diluted sample was aliquoted into a 2 mL Eppendorf tube. Phenol nitrile (400 µL) and alkaline hypochlorite (400 µL) were added to the sample and the standards, and the tube contents were mixed and allowed to sit for 30 min for colour to develop. The sample was read at 625 nm in the spectrophotometer. A standard curve of ammonia from 0 to 150 mmol/L was constructed.

Phenolic and indolic compounds: For *p*-cresol, indole and phenol analysis the caecal, colon (proximal and distal) and faecal samples were centrifuged at 4 °C for 10 min at 2500× *g*, and the supernatant diluted to 1:1 (500 µL + 500 µL) with HPLC-grade methanol directly in the autosampler vials. The samples were run on a Thermo Scientific Vanquish Horizon UHPLC using a Phenomenex Kinetex C18 (Phenomenex, Torrance, CA, USA) (100 × 2.10 mm, 1.7 µm) column at 40 °C. The mobile phase solvents were, A, 0.1% formic acid and, B, acetonitrile, with a flow rate of 300 μL/min. The gradient started at 30% B, increasing to 40% B at 3 min and to 95% B at 5.5 min, holding for 2.5 min. The injection volume was 1 µL. Detection was by a diode array detector set at 271 nm for phenol and indole and 279 nm for *p*-cresol. The spectra data (250–600 nm) were collected for confirmation. *p*-cresol, indole and phenol analytes were measured against authentic standards (1–100 µg/mL). Data were processed using the Chromeleon Chromatography Data System (Version 7.3.2; Thermo Fisher Scientific, Waltham, MA, USA) software.

### 2.5. Microbiome Effects

DNA was extracted from the dry faeces using NucleoSpin Soil kits (Macherey-Nagel, Düren, Germany) according to the manufacturer’s instructions. A sequencing library was prepared with the TruSeq Nano DNA LT Library Prep Kit. Pair-end sequencing was performed following standard protocols using the Illumina MiSeq platform with the MiSeq V3 600 cycle sequencing kit (Illumina, San Diego, CA, USA). Raw sequences were demultiplexed with 0 mismatches in the barcode sequences. Data processing was conducted using the DADA2 version 1.10 protocol to produce amplicon sequence variants (ASVs). Microbial richness was determined by analysis of the number of Observed Species, an index for α-diversity. β-diversity was determined using Bray–Curtis dissimilarity-based principal coordinates analysis in the microbiota of faecal samples from individual rats on all four diets. Data of the ASV table were analysed using MicrobiomeAnalyst version 2.0 (https://www.microbiomeanalyst.ca/ (accessed on 12 August 2024)), with the metagenomics prediction performed using the Tax4Fun module [20].

### 2.6. Faecal Bulking Analysis

Faecal collections were made daily during the balance period and the faecal pellets placed in plastic specimen pots, frozen at −20 °C, freeze-dried and weighed before determining their hydration properties. The water-holding capacity (WHC) of the faeces was measured as g water/g dry matter by accurately weighing about 1 g of the freeze-dried intact pellets into pre-weighed specimen pots as a single layer, adding enough water (about 5 mL) for the pellets to passively imbibe and leave a small surplus. After 24 h of imbibition the pellets were gravity drained for 20 min by placing the pots on a slope and aspirating excess water with a Pasteur pipette before reweighing. Rehydration for 48 h did not significantly increase the water uptake.

To calculate the faecal bulking capacity of the diets, the faecal dry matter output (FDM) was adjusted to faecal dry matter per 100 g feed ingested (FDM/100 g) by allowing for spillage and refusals, and the hydrated faecal mass, a model of the human faecal mass, calculated as follows:

Hydrated faecal mass/100 g diet = FDM/100 g diet + (FDM/100 g diet × WHC).

### 2.7. Faecal Polysaccharides

Freeze-dried faecal subsamples were bulked within each treatment and the polysaccharide content of each of the four bulked samples determined in duplicate from monosaccharide release by acid hydrolysis. Each sample (100 mg) was accurately weighed into a 12 mL glass culture tube, 1.5 mL of 72% (*w*/*w*) sulfuric acid added, and the tubes incubated at 35 °C for 60 min with frequent stirring to ensure complete dispersion of the sample. Water (8.5 mL) was added and the tubes placed in a hot (95–100 °C) water bath for 60 min. After cooling the tubes were centrifuged (2000× *g*, 10 min) to clarify. Sugars were determined in the hydrolysate supernatant using the dinitrosalicylic acid (DNS) reagent as follows: Hydrolysate (0.1 mL) was placed in a glass tube and 0.1 mL of 1M of NaOH added to approximately neutralise the aliquot before adding 0.8 mL of DNS reagent. The tubes were heated for 15 min in a boiling water bath and cooled, 4 mL of water was added, and the colour was read at 530 nm. The DNS reagent consisted of glucose (0.5 mg/mL): 4M of a NaOH: DNS stock solution mixed in the ratio of 1:1:5. The DNS stock solution consisted of 10 g of 3,5-dinitrosalicylic acid dissolved in 1.0 L of water containing 16 g of NaOH + 300 g Na-K tartrate and allowed to stand for 2 days before use.

The reference material taken through the whole hydrolysis and colorimetry procedure was ash-free cellulose filter paper (Whatman™, Kent, UK). Glucose standards were also included at the colorimetry stage.

### 2.8. Secondary Faecal Fermentation and Protein Putrefaction

For the in vitro secondary fermentation, rat faecal samples were freeze-dried, ground and pooled within each treatment to serve as substrates to be incubated with human faecal inoculum, with and without added meat protein. The substrates were weighed into fermentation tubes (0.30 g per tube; *n* = 6 per treatment). To half of the fermentation tubes (*n* = 3 per treatment), 0.30 g of beef protein was added. Dry beef protein had been prepared by acetone extraction of cooked beef mince.

Faecal inoculum was prepared from faeces donated by seven healthy human adults who had not taken antibiotics in the previous 3 months. The study was ethically approved by the Central Health and Disabilities Ethics Committee in New Zealand under protocol 13/CEN/144. A 20% *w*/*v* faecal slurry was prepared by mixing the faecal samples with anaerobic sterile 0.01 M phosphate-buffered saline (PBS; pH 7.2; Sigma-Aldrich, Auckland, New Zealand) with 10% *v*/*v* glycerol and 0.05% *w*/*v* cysteine within 30 min of collection [21]. The slurry was aliquoted and stored at −80 °C.

A pooled human faecal inoculum was prepared by combining equal proportions of thawed faecal slurries in an anaerobic Whitley A95HEPA Workstation (Don Whitley Scientific, Bingley, UK). A sterile carbohydrate-free basal medium (pH 7.0) was added to each fermentation tube [21], followed by the addition of the pooled human faecal inoculum to reach a final concentration of 1% faeces *w*/*v*. The fermentation was performed at 37 °C under anaerobic conditions with orbital shaking (Lab Companion CMS-350, Daejeon, Republic of Korea) at 100 rpm for 32 h. The final concentration of the rat faecal substrate was 1 g per 100 mL, and the beef protein was 1 g per 100 mL, if included. In addition to the rat faecal substrates, inulin (1 g/100 mL; fibre control) and no substrate (water control) were included in this experiment, with and without beef protein. Subsamples (2 × 1 mL) were taken just after inoculation (defined as 0 h samples) and at 8, 18, 24 and 32 h after inoculation. One aliquot was directly frozen at −80 °C. The other aliquot was centrifuged at 16,000× *g* at 4 °C for 5 min, and the supernatant and pellet were separated before storing them at −80 °C for later analysis of putrefaction products.

### 2.9. Data Analysis

Statistical analyses were conducted using the Genstat software (version 18, VNSi Ltd., Hemel Hempstead, UK). Faecal parameters, gut contents and putrefaction products were compared between diets with one-factor analysis of variance (ANOVA). Residual plots were checked for normality and homogeneity of variance. Faecal parameters were log-transformed to stabilise the variance before analysis. Comparisons between individual means were made using Tukey’s test.

The development of secondary putrefaction products over time was compared between diets with a repeated-measures ANOVA. Contrasts were fitted to calculate the difference between treatments at each time point.

For microbiome analysis 1-way ANOVA with Tukey’s post hoc was used to determine differences between groups. False Discovery Rate (FDR) was performed using the Benjamini–Hochberg method, controlling FDR at 0.05. Taxonomic data at the genus level were analysed with LEfSe (Linear discriminant analysis Effect Size) to identify discriminant microbial signatures, using the defined threshold of Linear Discriminant Analysis (LDA) > 2.

## 3. Results

### 3.1. Animal Feeding and Growth

The rats in all groups consumed most (80%) of their 100 g allocation of food across the 4-day balance period, and intakes were similar in all diet groups (Casein 18.6%: 79.1 ± 8.1 g. Casein 37.2%: 80.8 ± 7.9 g. Meat: 80.2 ± 6.7 g. Pea: 79.6 ± 6.7 g (mean ± SD)). Growth of the rats in all groups was also steady and similar over the 9-day trial period (Casein 18.6%: 36.8 ± 12.9 g. Casein 37.2%: 48.0 ± 8.2 g. Meat: 46.2 ± 10.4 g. Pea: 43.0 ± 6.4 g (mean ± SD), and all rats maintained good health. Samples were obtained from all animals if available. In a few animals some gut regions, predominantly the colon, contained insufficient material for some analyses because it was distributed discontinuously, especially in the case of faecal pellets in the rat colon. Missing points were allowed for in analysis.

The animals were monitored closely and did not show any signs of stress due to the need to ingest powdered diets. The diets for all groups were of similar consistency, all rats consumed nearly all their daily allocation, and all grew equally well, so there was no reason to believe that stress was a confounding factor in the study.

### 3.2. Faecal Bulking and Hydration

Faecal dry matter outputs adjusted to 100 g feed intake by rats fed both casein diets and the meat diet were very similar but were less than half the output of rats fed the pea diet (Table 2 and Supplementary Figure S3). The faeces of the pea-fed rats had, on average, a substantially (66%) larger water-holding capacity than those of the casein and meat-fed rats (Table 2 and Supplementary Figure S4), which were similar to one another. Combining faecal dry matter output per 100 g of diet and water-holding capacity accentuated the difference between the pea and the three other diets and showed that pea induced about 3.5 times the hydrated faecal mass of the other diets on an equal-feed-intake basis (Table 2 and Supplementary Figure S5). The pattern of differences in cumulative hydrated faecal bulk during the 4-day balance period was closely reflected in the mass of contents in the caecum, proximal colon and distal colon of the rats at the time of euthanasia (Table 3).

### 3.3. Protein Putrefaction Products

**Figure 1:**
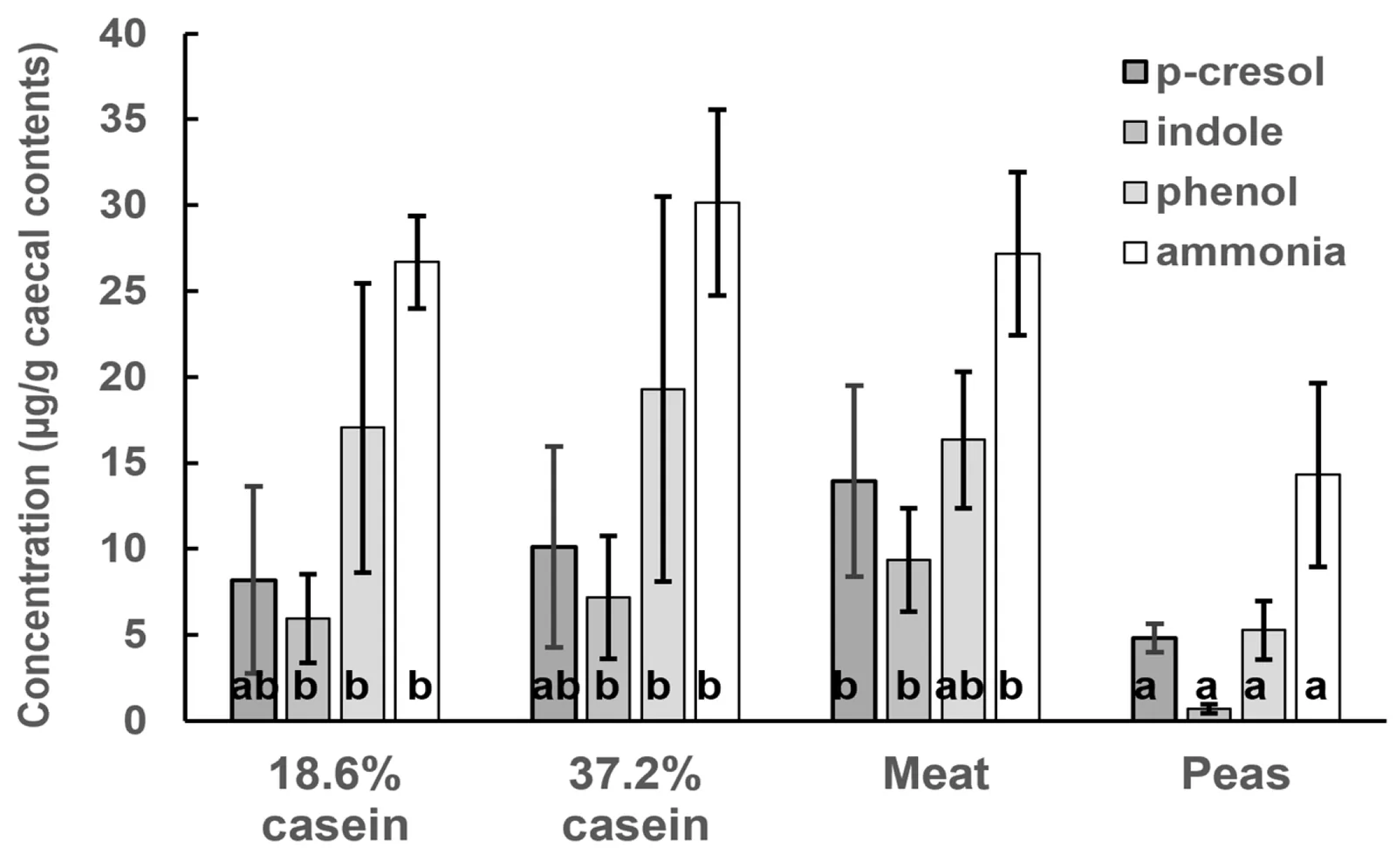
Putrefaction product concentrations in the caeca of rats fed 18.6% and 37.2% casein, 18.6% meat-protein, and 18.6% pea-protein diets, with the pea protein in the form of pea cotyledon (means ± SD). Bars with a different letter within an analyte differ significantly, based on Tukey’s test: *p*-cresol, F = 4.3, *p* = 0.014; indole, F = 8, *p* < 0.001; phenol, F = 5, *p* = 0.007; ammonia, F = 18, *p* < 0.001 (Supplementary Table S1).

The concentrations of all protein putrefaction products measured in the caecal contents—ammonia, indole, *p*-cresol, and phenol—were inversely related to the faecal mass. The putrefaction product concentrations in caecal contents of rats fed the pea diet were significantly (*p* < 0.001) and substantially lower than in rats fed the other diets, which differed very little from one another (Figure 1; Statistical analysis in Supplementary Table S1). For instance, ammonia was 50% lower in the pea diet than in the 18.6% casein diet. Furthermore, this pattern of differences between diets was maintained throughout the hindgut—caecum, proximal colon and distal colon (Supplementary Figure S6)—despite large variation in faecal mass in the colonic regions (Table 3).

### 3.4. Caecal Short-Chain Fatty Acids

**Figure 2:**
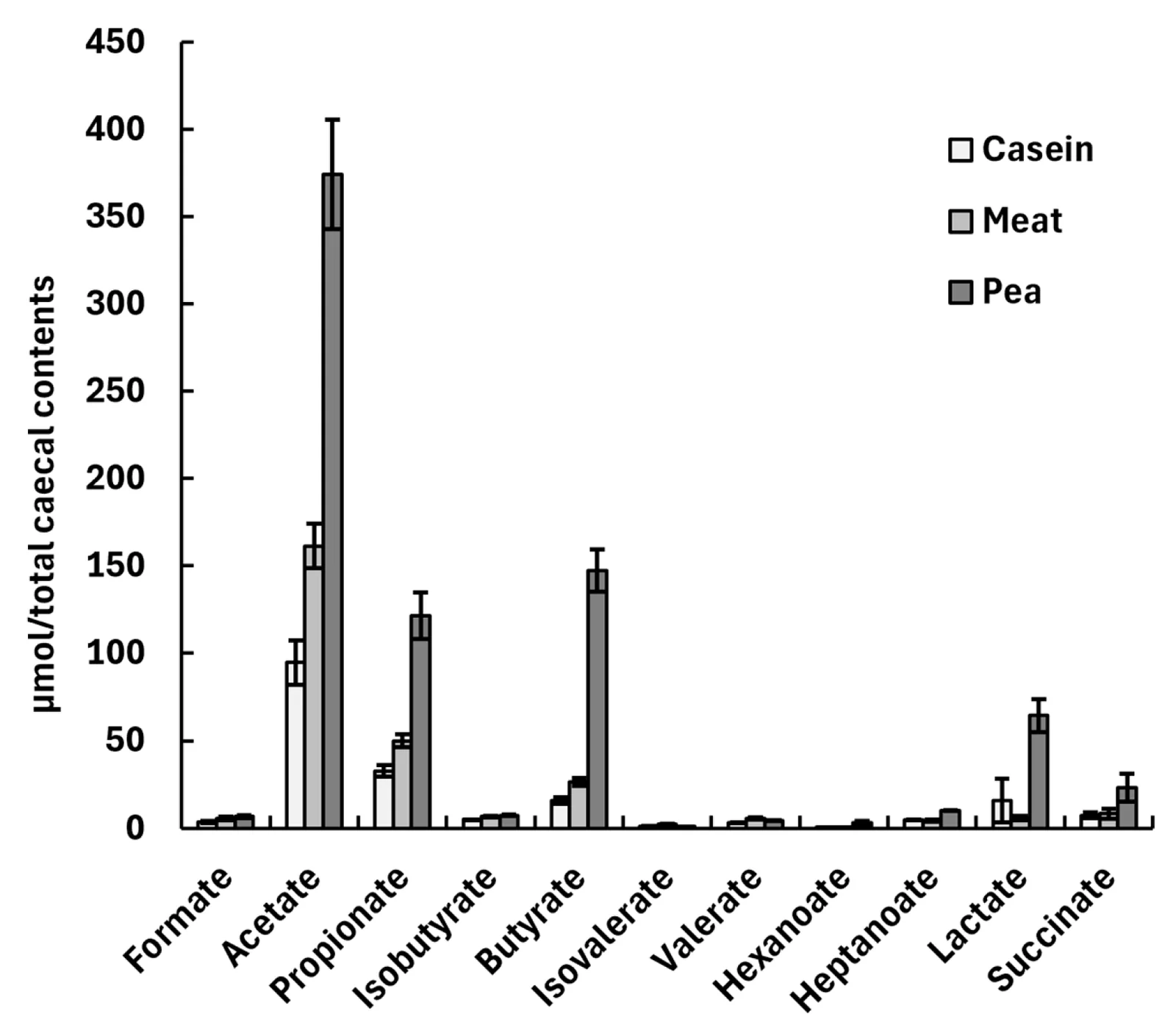
Short-chain fatty acid load in caeca of rats fed casein, meat and pea diets, each containing 18.6% protein at time of sacrifice. Estimated by multiplying concentration (Table 4) by the mean mass of caecal contents (Table 3).

Short-chain fatty acid (SCFA) concentrations were significantly higher in caecal contents from the pea-fed than from the casein- and meat-fed rats for most fatty acids (Table 4). When adjusted for the volume of caecal contents at the time of sacrifice, the large difference between the pea-fed and other groups was greatly accentuated (Figure 2). Butyrate and lactate were notably greater in the pea-fed than in other groups in the ratio of about 7:1 and 4:1 respectively, whereas the average ratio for the major SCFA, acetate, was 1.4:1, and for total SCFAs the ratio of pea to mean of other groups was 1.6:1.

### 3.5. Faecal Microbiota

**Figure 3:**
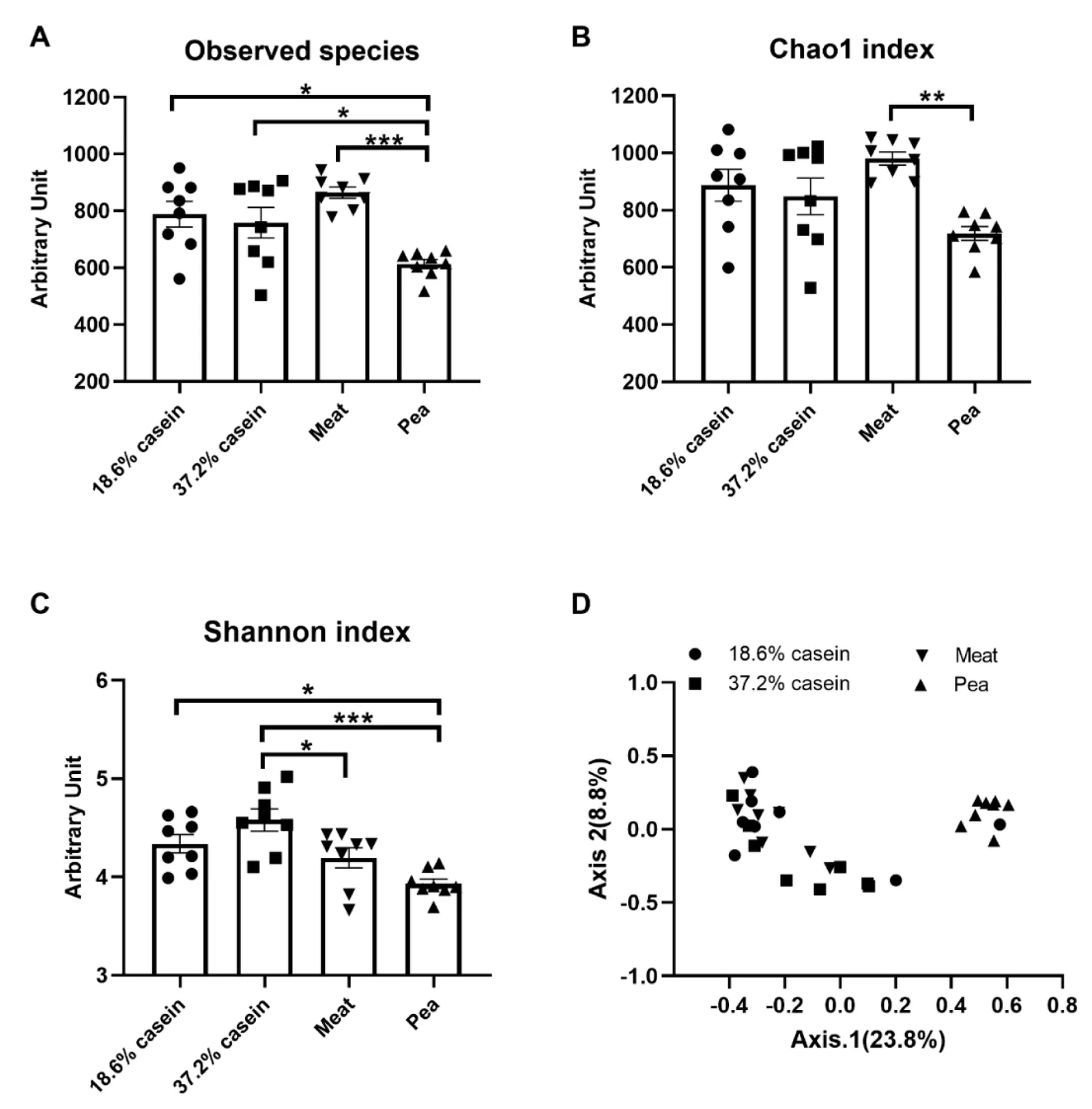
Gut microbiota diversity measured in the faeces of rats fed 18.6% casein, 37.2% casein, 18.6% meat-protein and 18.6% pea-protein diets. * *p* < 0.05, ** *p* < 0.01, *** *p* < 0.001. (**A**) Observed species; (**B**) Chao1 index. (**C**) Shannon index. (**D**) principal components analysis.

**Figure 4:**
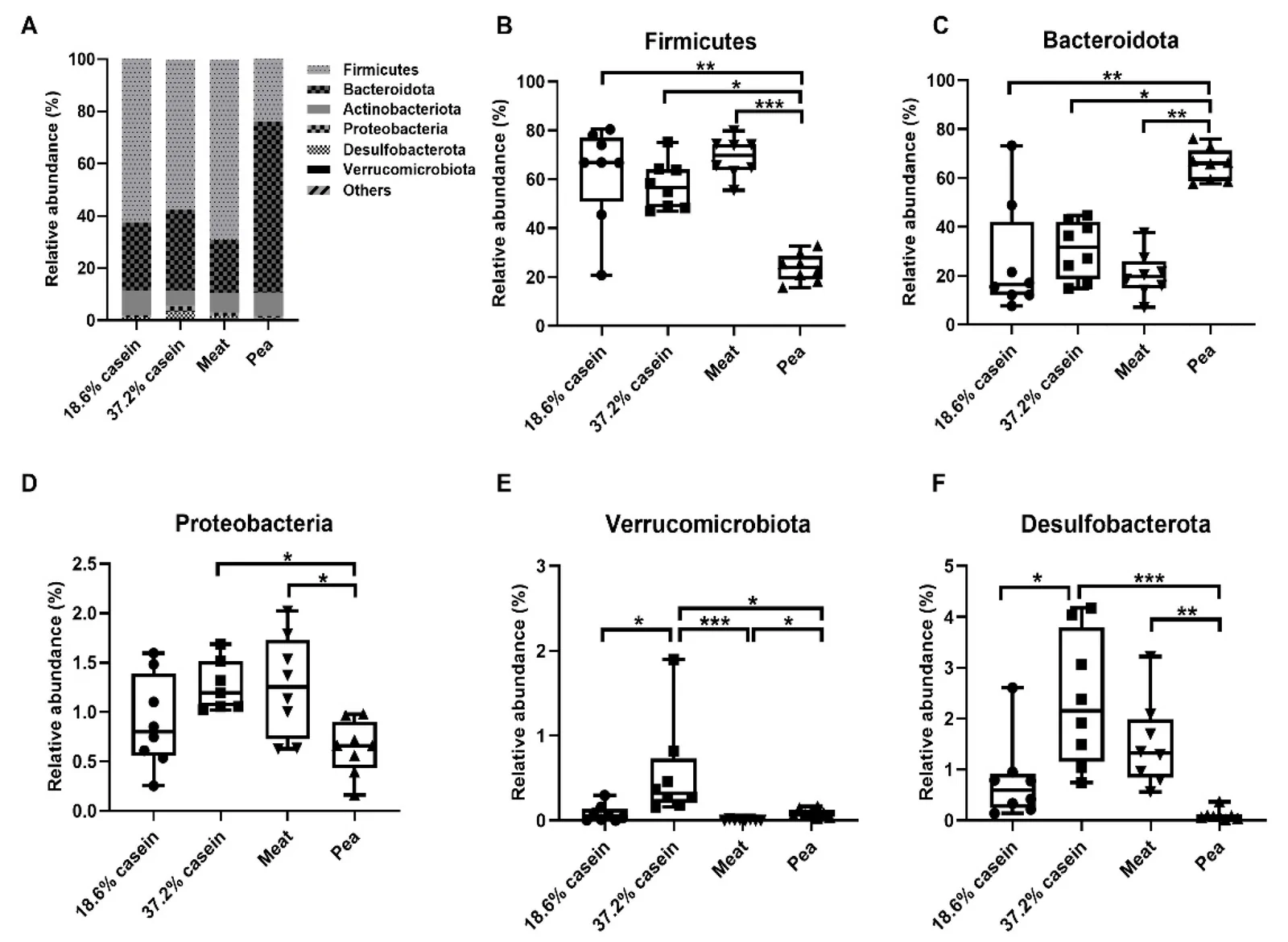
Gut microbiota composition at the phylum level measured in the faeces of rats fed 18.6% casein, 37.2% casein, 18.6% meat-protein and 18.6% pea-protein diets. (**A**) Relative abundance; (**B**–**F**) sources of significant difference. * *p* < 0.05, ** *p* < 0.01, *** *p* < 0.001.

**Figure 5:**
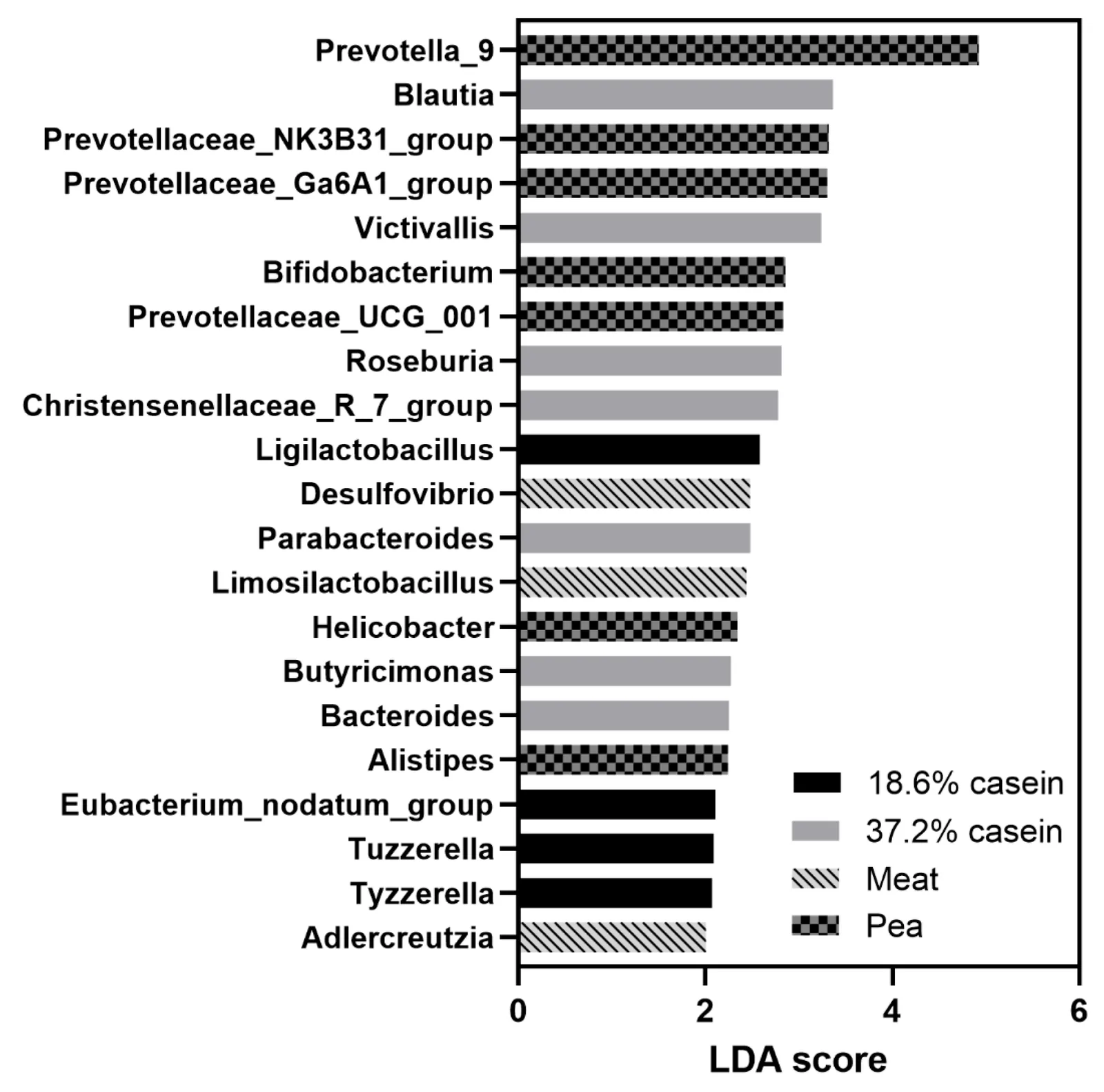
Gut microbiota composition at the genus level measured in the faeces of rats fed 18.6% casein, 37.2% casein, 18.6% meat-protein and 18.6% pea-protein diets.

The pea diet showed the lowest α-diversity of the four diets, being significantly lower in microbial richness than the casein and meat diets (Figure 3A–C). It also showed a distinctive microbial structure in principal coordinates analysis (Figure 3D), with substantial differences between the meat and high-casein (37.2%) diets at the phylum (*p* < 0.001) (Figure 4) and genus (*p* < 0.05) levels (Figure 5). At the phylum level, Firmicutes and Bacteroidota were the dominant phyla in the microbiota. The pea diet reduced the relative abundances of Firmicutes (Figure 4B), Proteobacteria (Figure 4D) and Desulfobacterota (Figure 4F) while increasing those of Bacteroidota (Figure 4C). The high-protein diet significantly increased the relative abundance of Verrucomicrobiota (Figure 4E). At the genus level, the high-protein diet enriched Blautia, Victivallis, Roseburia, Parabacteroides, and Bacteroides; Pea protein enriched the Prevotella_9 group, Bifidobacterium, and Alistipes; while meat protein enriched Desulfovibrio and Limosilactobacillus (Figure 5).

**Figure 6:**
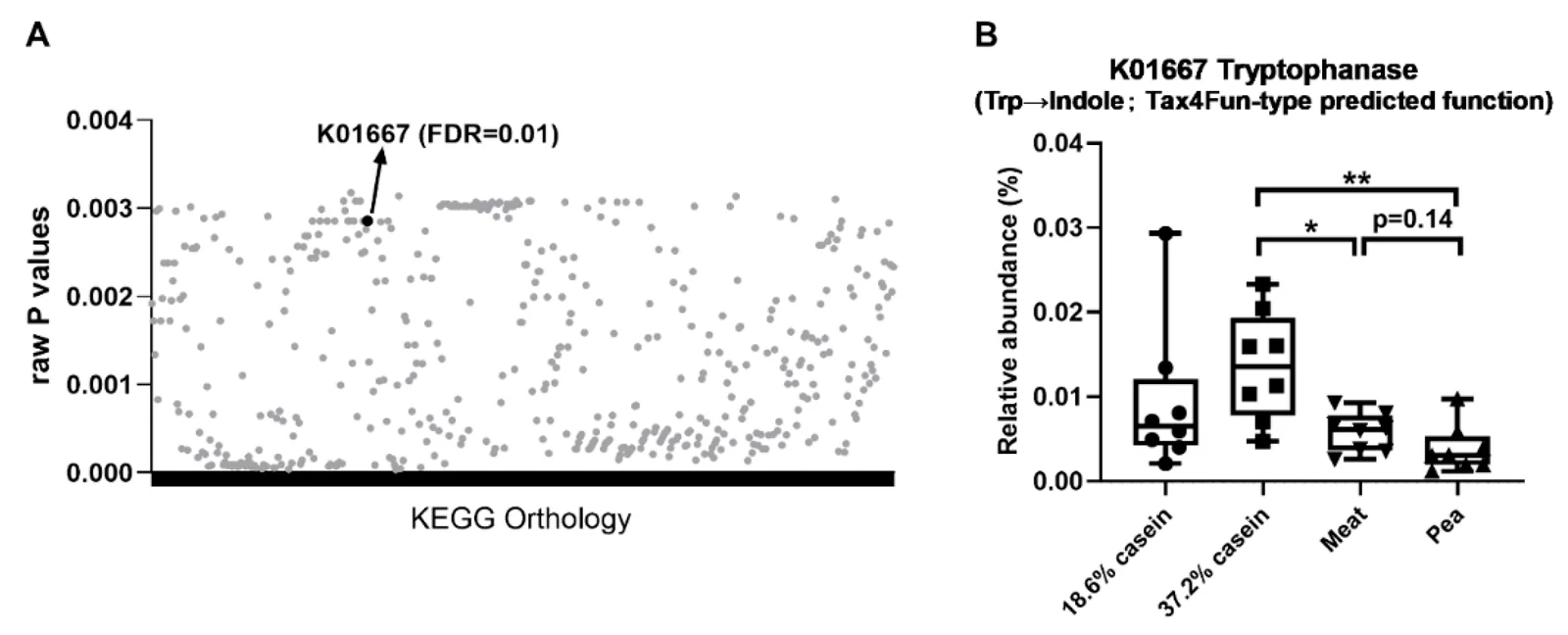
Tax4Fun-type Metagenomics prediction of microbial function measured in the faeces of rats fed 18.6% casein, 37.2% casein, 18.6% meat-protein, and 18.6% pea-protein diets. (**A**) Statistical analysis of all KEGG orthologies. (**B**) K01667 related to tryptophanase based on the Tax4Fun prediction. * *p* < 0.05, ** *p* < 0.01.

Metagenomic prediction analysis showed that indole synthesis may be upregulated in the high-protein (37.2% casein) diets and that it may be greater in the meat- than pea-protein diet (Figure 6), consistent with the results in Figure 1.

### 3.6. Secondary Faecal Fermentation

**Figure 7:**
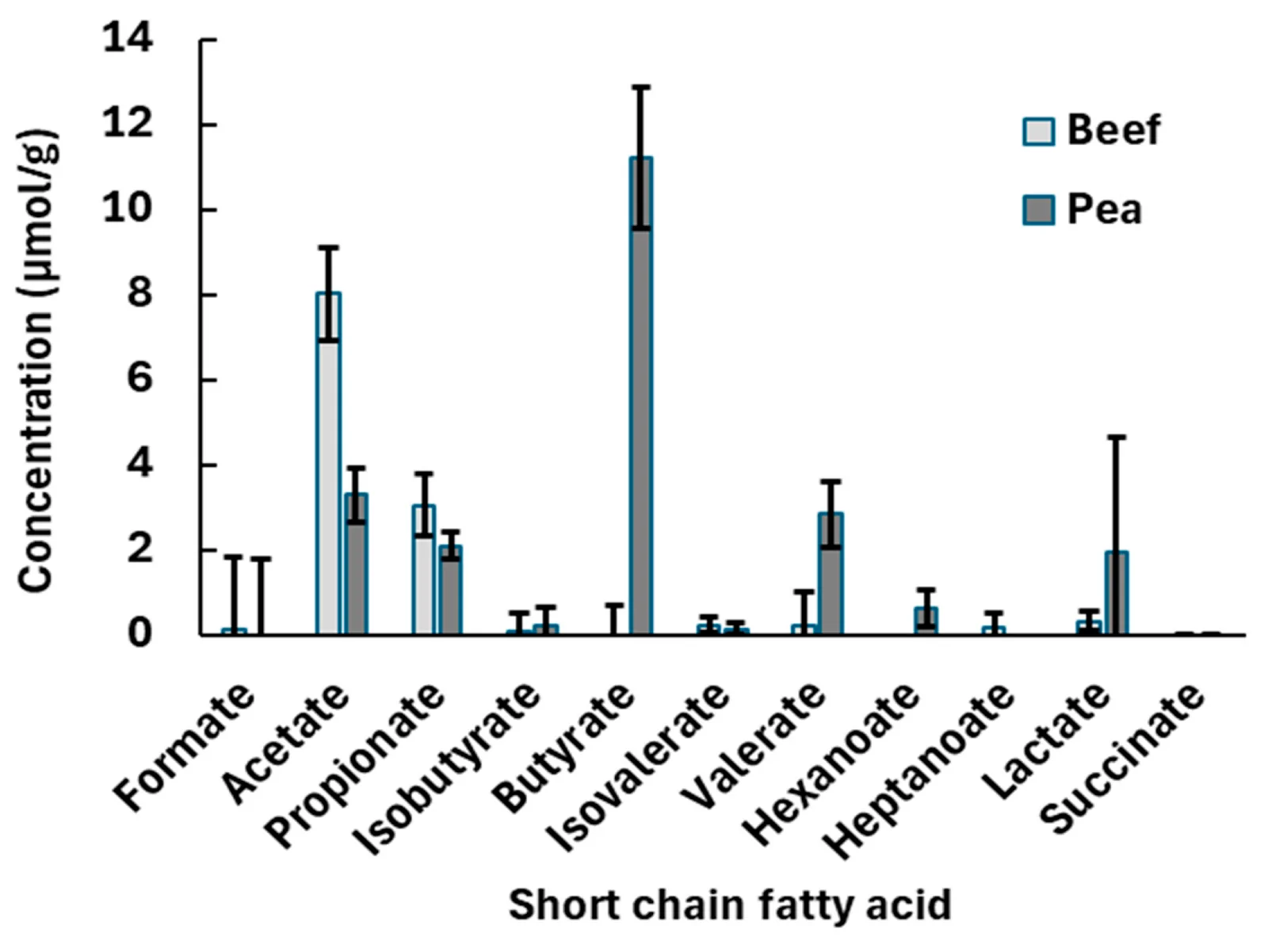
Short-chain fatty acid production increase over water control after 32 h of secondary fermentation of faeces from rats fed diets containing 18.6% protein from meat or 18.6% protein from pea cotyledon, using human faecal inoculum.

**Figure 8:**
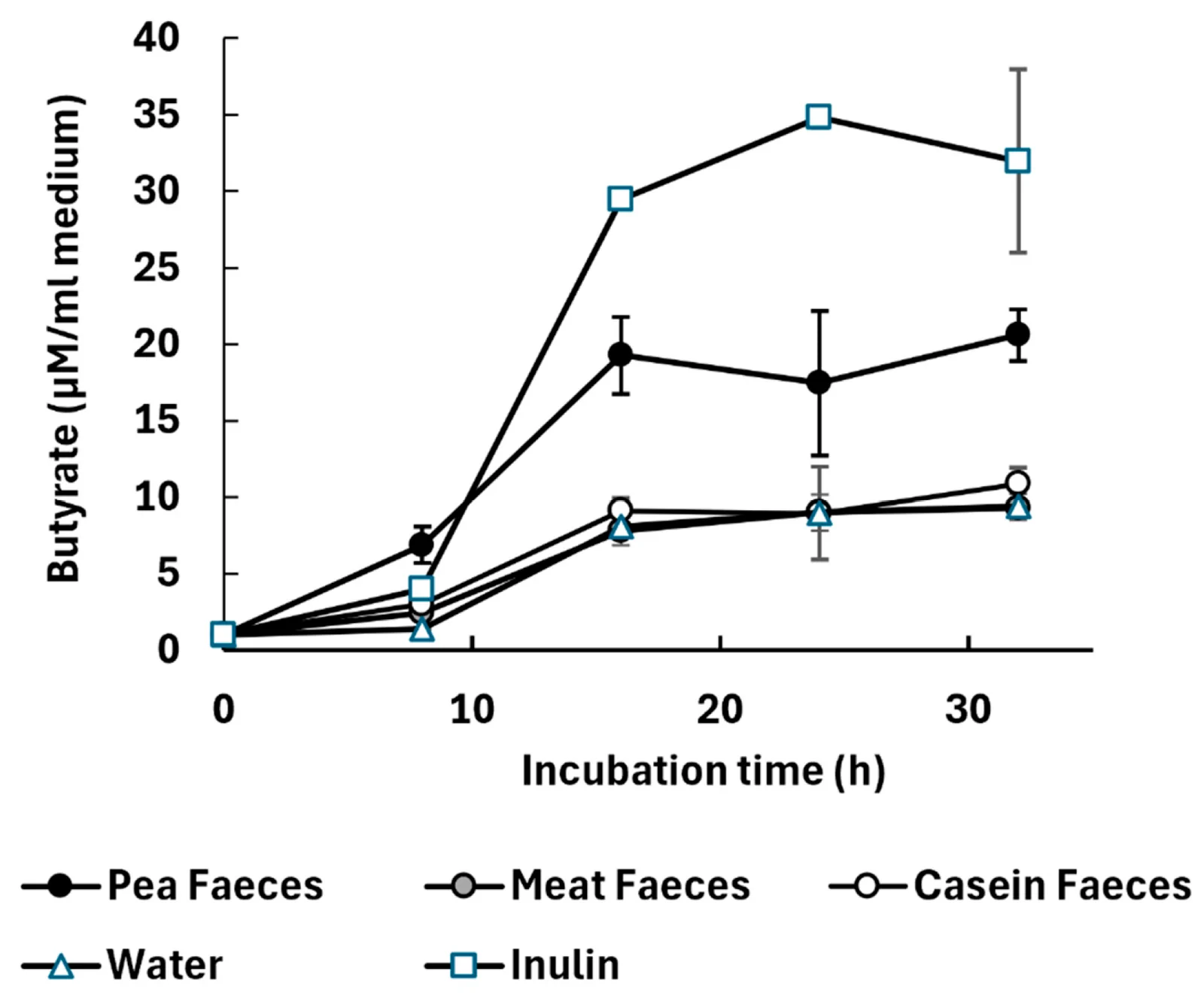
Butyrate production during secondary fermentation of faeces from rats fed diets containing 18.6% protein from meat, 18.6% protein from pea cotyledon, water (negative control), or inulin (positive control) (means ± SD).

Faeces from pea-fed rats induced a greater (*p* < 0.001) increase in butyrate above the water control than the faeces from casein and meat-fed rats during secondary fermentation with human faecal inoculum (Figure 7: shows data at 32 h of fermentation), with a steady increase in butyrate concentration over the course of fermentation (Figure 8). In contrast, butyrate production from faeces of the meat-fed rats was no greater than from the water controls. The greatest butyrate production was achieved in the inulin positive control. The observed trends in butyrate production were similar in the presence and absence of added protein.

**Figure 9:**
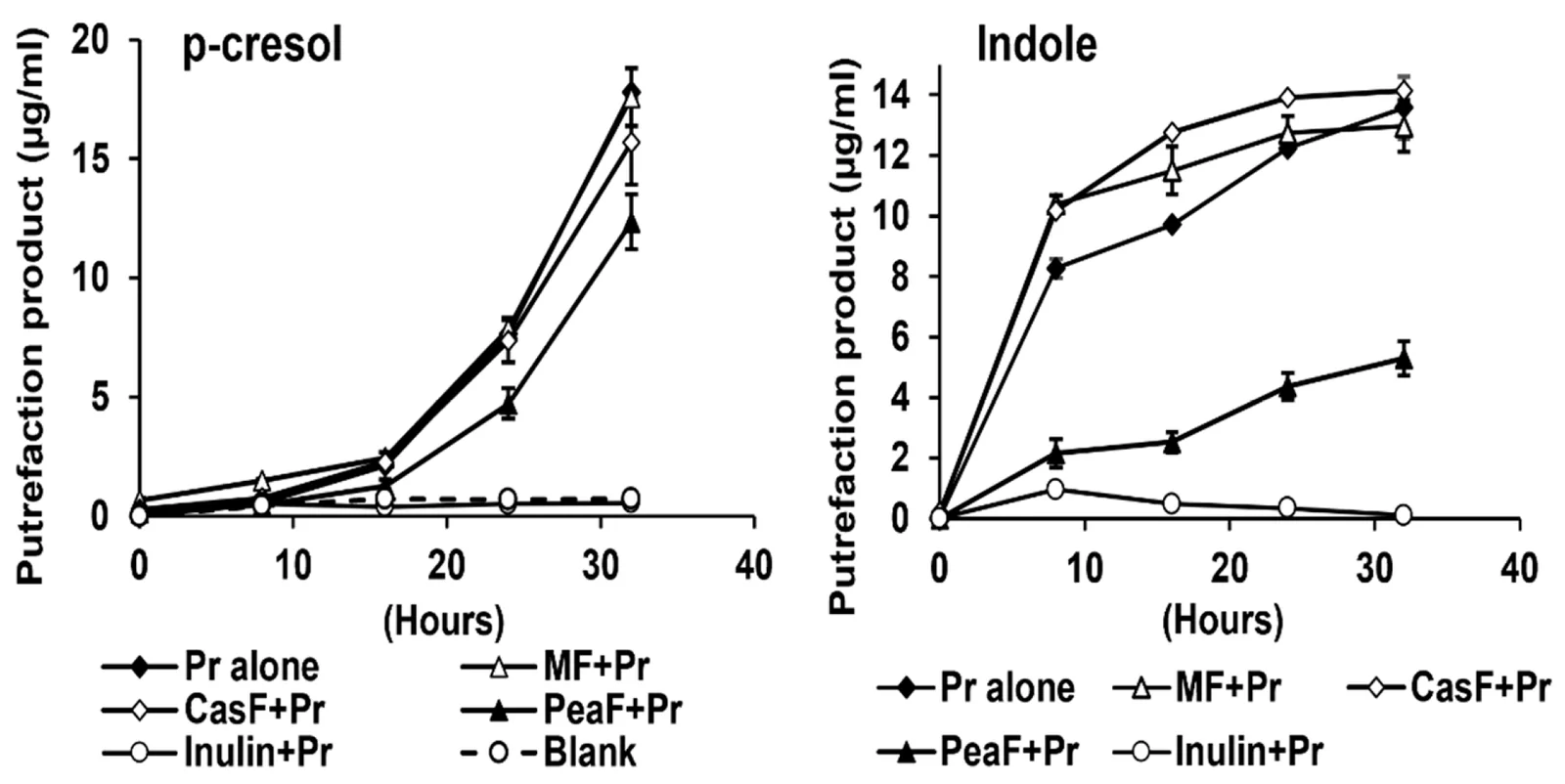
Effect of inulin and faeces from rats fed meat (MF) and pea diets (PF) on the generation of *p*-cresol and indole during fermentation of protein (Pr) with human faecal inoculum (means ± SD). The blank contained no added faeces or protein. Statistical analysis presented in Supplementary Tables S2 and S3.

The generation of protein putrefaction products during fermentation of protein by human faecal inoculum was affected differently by adding inulin, dried rat faeces from 18.6% meat-protein diet, or faeces from the diet containing 18.6% protein from pea cotyledon. Inulin completely suppressed production of putrefaction products, faeces from the pea-fed rats partially suppressed *p*-cresol and indole production (Figure 9), while faeces from the meat and casein diets had no inhibitory effect.

The putrefaction products, indole, *p*-cresol, and phenol during secondary fermentation of faeces from all diets, with and without added protein, after 0, 8, 16, 24, and 32 h of fermentation are shown in Supplementary Tables S2–S4.

### 3.7. Faecal Non-Digested Polysaccharides

All faecal samples contained almost the same proportion of non-digested polysaccharides (NDPs) (Supplementary Table S5; average of means, 36.4%; SD, 0.73; range, 1.47%), but because faecal output was greater for the pea than for the other diets, the total faecal NSP output was correspondingly greater for the pea diet. But based on the intakes of dietary fibre by the rats, the theoretical recoveries of fibre as faecal NDPs for the 18.6% casein, 37.2% casein, meat and pea diets were 62.4, 66.1, 66.6, and 26.4%, respectively (Supplementary Table S5), reflecting fermentation of the pea NSP.

The proportion of apparent NDPs was almost the same across all faecal samples. All diets also had the same base content of dietary fibre mixture (2.93%) and the same protein and fat contents. Because of this it was possible to estimate (by difference), taking into account the differences in faecal dry matter output per 100 g of diet, that about 53% of the NDPs in the faeces from the pea diet were from pea. This represented a maximum of about 26% apparent survival of pea dietary fibre in the faeces (Supplementary Figure S7).

## 4. Discussion

The experiments reported here have shown how several complementary factors providing protection against protein putrefaction products may act concurrently when legume cotyledon, rather than meat, is used as a source of protein. These factors may all be attributed to the properties of undigested carbohydrate (dietary fibre) in the hindgut. They include hydration and bulking throughout the gastrointestinal tract, with consequent dilution of protein putrefaction products, a hindgut microbiome in the pea-fed animals that was functionally adapted for carbohydrate fermentation rather than protein putrefaction, increase in the quantity and proportion of protective short-chain fatty acids (especially butyrate), distal translocation of fermentable carbohydrate to the extent that it appears in high proportion in faeces, and protection against putrefaction of colonic protein by dietary carbohydrates that have survived gut transit, as revealed in the secondary fermentation. Additionally, an inevitable result of increased intestinal bulk will be an increased transit rate, reducing the residence time during which putrefaction could occur, the duration of exposure of the host gut to the products of putrefaction, and the time available for fermentable polysaccharides to become depleted [6]. Dietary fibre and protein were not independent variables in the study design, which involved comparing pea with animal protein sources, but they emerged as the components having the most influence on the study outcomes.

Differences between the pea and the 37.2% casein diet were almost identical to those between the pea and 18.6% casein diets, further suggesting that the presence of dietary fibre was a far more important factor than dietary protein loading per se in the present study. If anything, a protein encapsulating effect of pea cotyledon cell walls might have resulted in greater colonic protein loading than in the casein and meat diets, yet the protective effect of pea was dominant, and protein putrefaction products were no greater in the pea than in other groups. Furthermore, the similarity in the growth rate of all groups suggests similar protein availability in all groups with no greater loss of protein to the colon in the pea compared with other groups.

The focus of this research was to compare the colonic effects of meat and pea cotyledon as protein sources in diets formulated to provide an approximately equal protein dose. The diets were intentionally not balanced with respect to dietary fibre content to allow for intrinsic differences between the protein sources, pea cotyledon and meat, to emerge. With the diet formulation used, and assuming dry peas contain 20% total dietary fibre as a conservative estimate, the pea diet (800 g/kg of diet) would have provided about 140 g/kg more dietary fibre than the meat, although all diets contained a small mixed-fibre component (Table 1) to maintain a basal microbiome. As the results show, the pea cotyledon provided short-chain fatty acids from fermentation, such as butyrate, which has been shown to have multiple benefits for colonic health [22]. Furthermore, biomass resulting from fermentation-supported bacterial growth, in association with unfermented fibre, will have contributed to the much greater hydrated faecal mass from the pea than from the non-pea diets.

Increased hydratable bulk throughout the gastrointestinal tract was one of the clearest differences between the pea cotyledon and other diets. Based on measurements during faecal collection this resulted from an increase in both digestion-resistant dry matter and its increased water-holding capacity, both significantly greater in the pea-fed diet than in the other diets (Table 2), and typical properties of unfermented plant cell wall remnants in the gut [23]. The results from the experiments reported here show the valuable protective role of dietary fibre due to both its physical bulking effects revealed in the model of human hydrated faecal mass and its role in butyrate biosynthesis in the colon.

The increase in tryptophanase gene expression in the high-protein (37.2% casein) and meat diets relative to pea, revealed by metagenomic analysis, suggested a functional adaptation of the microbiome to cope with low carbohydrates in the presence of available protein substrates. Tryptophanase is a bacterial enzyme in the catabolism of L-tryptophan to indole and ammonia [24], so it may have an important role in linking colonic protein putrefaction to CRC risk.

In the pea-diet group, increased faecal water-holding capacity accompanied low microbial richness. Another recent study of the gut fermentation of plant-based food materials found a negative correlation between water-holding capacity and α-diversity, as reflected in numbers of observed genera [25]. Although the mechanism underlying the correlation remains unknown, the decreased α-diversity may serve as an adverse marker of the gut microbiome and reflect reduced microbial resilience or be a diet-artefact specific to pea fibre intake. One possible reason is that a non-starch polysaccharide enriched in a pea diet is degraded by a limited group of microbes, mostly within Bacteroidota [26]. A relatively high hind-gut loading of fermentable polysaccharides in the split pea group may have created a disadvantageous microenvironment for the survival of microbes unable to utilise the substrate, leading to low microbial diversity. Also, the dominance but limited diversity of specialist polysaccharide fermenters in the pea diet may have represented a functional adaptation to the limited diversity of the non-digestible polysaccharides in the diet, which, nonetheless, resulted in a flush of short-chain fatty acids beneficial to the host. Non-digestible dietary polysaccharides in the meat and casein diets, on the other hand, would have been mostly from the more diverse but less abundant basal mixture of apple, potato and wheat fibres, with additional endogenous mucopolysaccharides. The results suggest that increased α-diversity *per se* is not advantageous unless it represents microbial adjustment to a correspondingly diverse but also abundant substrate matrix, as would be expected in a mixed diet of free-living humans, rather than in the formulated experimental diets used in the present confined study. In other words, the lack of resilience attributed to low α-diversity could result from functional adaptation to an inadequately diverse colonic fibre load.

The results from the analysis of putrefaction products—ammonia, *p*-cresol, indole, and phenol—in the caecal contents (Figure 1) confirm the importance of colonic bulk as a diluent of protein putrefaction products that may contribute to protection against CRC. While the total amount of putrefaction products in the caecal contents was almost the same for all four diets, because of the greater caecal bulk in the split pea-fed rats, their concentration was about half that of the meat and casein diets (Supplementary Figure S6). Combining the dilution effect with a reduced residence time, as an unavoidable result of increased gastrointestinal bulk, would reduce the degree of exposure of the colonic epithelium to the putrefaction products.

NDP analyses showed that faeces from all four diets contained the same proportion of NDPs, although the high apparent recovery of fibre from the non-pea diets (average 65.0 ± 2.3%) may have included a contribution from endogenous and/or bacterial polysaccharides. Nonetheless, because the proportion of apparent NDPs in faeces was the same for all diets, and all diets had the same basal 2.93% dietary fibre content, knowing the faecal dry matter per 100 g of diet allowed us to estimate that about 54% of the NDPs in faeces from the pea diet were of pea origin, and this represented about 13% of the ingested pea fibre and constituted 19% of faecal dry weight (Supplementary Table S5). This amount of pea polysaccharides in the faeces, combined with the secondary fermentation data, shows that the pea fibre was fermented internally, and the supply of it for colonic fermentation was more than adequate. The label and determined dietary fibre contents (25.4% vs 21%) were in reasonable agreement in light of possible batch variation and typical inter-laboratory differences in fibre values, and the differences would not have affected the outcome of the study in view of the dominant effect of the pea fibre.

While it is certain that the pea fibre had been extensively but not completely fermented in the rat gut, the exact fibre loss is difficult to gauge because the contribution of bacterial and endogenous polysaccharides to faecal NDPs could not be accurately determined. So, in fact, probably less than 50% of the NDPs in the faeces from the pea were of pea origin. However, as the results of secondary fermentation show, enough remained in the faeces to function as an effective substrate. Faeces from pea supported secondary fermentation, whereas faeces from the meat (Figure 8) and casein diets were no better than the water control at stimulating short-chain fatty acid production and suppressing protein putrefaction. This suggests that the NDP content of faeces from the casein and meat diets was either exhausted NDPs or inert as a bacterial substrate, whereas the pea faecal NDPs remained a viable substrate for colonic fermentation.

Secondary fermentation of the rat faeces with human faecal inoculum with and without added protein was a novel and informative aspect of this study, and it non-invasively revealed the protective effect of diet at the distal level. Although effects of individual parameters may be known, it is uncommon that multiple effects, especially including bulking effects [27], are considered in combination.

The research described here was designed as a preliminary study of some fundamental differences between meat and legume cotyledon as protein sources, in the context of the EAT-Lancet Commission’s recommendation to move from meat to plant-based diets to reduce the incidence of non-communicable diseases, such as CRC, and to reduce the impact of livestock on environmental health. However, it should not be inferred from the present results that meat protein is less healthy than plant protein, but rather that protein in the presence of adequate dietary fibre is healthier than protein consumed without adequate fibre. As most of the benefits of the pea cotyledon diet seen here could be attributed to its dietary fibre content, one can argue that meat is no less beneficial than plant protein if it is consumed in a diet containing adequate dietary fibre. Moreover, as this study has shown, the dietary fibre should have an appropriate balance of properties—fermentability and colonic bulking.

Further research would be appropriate to show the safety of meat in the presence of sources of dietary fibre with an adequate balance of functional properties. Such properties would include polysaccharide fermentation throughout the large intestine, sufficient non-fermented bulk to ensure distribution of fermentation distally, and suppression of colonic protein putrefaction. Enough hydrated bulk should reach the distal colon to induce regular evacuation, leading to replenishment of fermentable polysaccharides and, importantly, host comfort. The spectrum of properties required for colonic health are all provided by the plant cell wall remnants remaining after foregut digestion of a diet based on fruit, vegetables and cereals.

The simultaneous measurement of several parameters that are dependent on various dietary fibre properties is a strength of the present study because it demonstrated the importance of being able to define different classes of dietary non-starch polysaccharides in terms of health-relevant functional attributes. Crude terms such as “soluble fibre” and “insoluble fibre” may give a very inaccurate indication of the health benefits of fibre sources when they do not represent either the effects of the fibre sources on health outcomes or their properties at the gastrointestinal location where they have their greatest impact on health. Food choices for health could be assisted by the development of food data that represent beneficial functional effects, such as hydrated faecal mass, distal fermentability, and water-holding capacity, for instance, which cannot be accurately expressed as soluble and insoluble fibre.

The results of the present study are likely to provide a valid/accurate representation of the relative effects of meat and pea cotyledon in the human gut. The rat is a hind-gut fermenter, like humans, in which the properties of the gut contents will be determined largely by the combination of food remnants that have survived foregut digestion and hindgut fermentation, the associated bacterial biomass, and water held in the faecal matrix. The microbiota in rats and humans is similarly diverse, with a similar functional capacity to consume most potentially fermentable material that reaches the hindgut [28,29], and the water-holding capacity of human and rat faeces is similar [14], making hydrated rat faeces an accurate indicative model of effects of diet on bulking in the human distal colon and stool.

These results support the importance of maintaining an adequate intake of mixed function dietary fibre that satisfies the need for both colonic fermentation and bulk in diets that are rich in protein. In practical terms this can be achieved by combining foods that provide fermentable primary cell walls, such as the parenchyma cells of fruit and tender vegetables, with sources of more fermentation-resistant secondarily thickened cell walls involved in plant structure and protection, including cotyledon cell walls, husks, vegetable tissue fibres, fruit skins and bran. In the case of legumes such as the yellow split pea used in the present study, the total dietary fibre load may have been due not only to robust cotyledon cell walls but also to the resistant starch they encapsulated [30].

### Limitations and Attributes of the Study

The present study has several limitations in terms of directly extrapolating the results to free-living humans under contemporary circumstances, as it was based largely on responses of restrained animals on diets not resembling human food intakes. The diets did not and were not intended to show the effect of different protein sources in a balanced diet, that is, in a diet providing an adequate intake of dietary fibre with a favourable balance of properties—fermentability, non-fermentability, and hydration of the non-fermented component. But the study is valuable in highlighting some of the variables that should be included in studies of diet-driven changes in the balance of colonic processes mediating risk factors for CRC in humans, including distal colonic bulk, microbiome functionality, substrate type and quantity, transit rate, and dietary fibre properties, including survival of a fermentable fraction into the distal colon.

There may have been some underlying bias because of the incomplete set of samples due to the lack of gut fill in some animals from the non-pea groups. However, low values rather than no sample would have accentuated the dominant effect of pea cotyledon fibre in the effects observed.

As mentioned, the 9-day adaptation period was probably insufficient for complete adaptation of the animals and gut microbiota to the different diets. It would be very interesting to extend this research by using more realistic diets, a longer adaptation period, and more direct markers of colon cancer risk.

## 5. Conclusions

Dietary fibre associated with dietary protein in legume cotyledon may play an important role in protecting against the harmful effects of protein putrefaction in the colon by several mechanisms: suppressing protein putrefaction, diluting protein putrefaction products, reducing the time of exposure to them, and the production of butyrate. Adequate intakes of plant dietary fibres and carbohydrate-based structures that contribute to both distal colonic fermentation and faecal bulk are likely to be important components of dietary protection against the products of colonic protein putrefaction.

## Figures and Tables

**Table 1:** Composition of trial diets (g/kg).

Component	1 18.6% Casein	2 37.2% Casein	3 Meat	4 Pea
Oil (Corn)	40	40	40	40
Vitamins ^1^	50	50	50	50
Minerals ^2^	50	50	50	50
Basal fibre mix ^3^	20	20	20	20
Beef fat	40	40	0	40
Starch	614	428	614	0
Casein	186	372	0	0
Meat (including fat)	0	0	226	0
Pea cotyledon	0	0	0	800
Total	1000	1000	1000	1000

^1^ Vitamin mix: contained the following components: (mg/kg diet) retinol acetate 5.0, DL-α-tocopheryl acetate 100.0, menadione 3.0, thiamin hydrochloride 5.0, riboflavin 7.0, pyridoxine hydrochloride 8.0, D-pantothenic acid 20.0, folic acid 2.0, nicotinic acid 20.0, D-biotin 1.0, myo-inositol 200.0, choline chloride 1500.0; (μg/kg diet) ergocalciferol 25.0, cyanocobalamin 50.0. ^2^ Minerals: Mixture contains the following components: (g/kg diet) Ca 6.29, Cl 7.79, Mg 1.06, P 4.86, K 5.24, Na 1.97; (mg/kg diet) Cr 1.97, Cu 10.7, Fe 424.0, Mn 78.0, Zn 48.2; (μg/kg diet) Co 29.0, I 151.0, Mo 152.0, Se 151.0. Contained 185.8 g/kg of cellulose (qv) contributing 9.29 g of cellulose per kg of diet. ^3^ Basal fibre mix: Contained equal quantities of apple fibre (VITACEL Apple fiber AF 401, J. Rettenmaier & Sohne GmbH & Co. KG, Rosenberg, Germany), potato fibre (J. Rettenmaier & Sohne GmbH & Co. KG, Rosenberg, Germany), and wheat fibre (VITACEL wheat fiber WF 600, J. Rettenmaier & Sohne GmbH & Co. KG, Rosenberg, Germany) (1:1:1). Together with cellulose in the mineral mix gives a total of 29.3 g of non-starch polysaccharide per kg of diet. Diet 1: 18.6% protein control. Diet 2: 37.2% protein (control × 2). Diet 3: Meat, included to provide 18.6% protein in diet. Diet 4: Split pea, included to provide 18.6% protein in diet.

**Table 2:** Properties of faeces from rats fed diets containing four different protein sources (means ± SD, *n* = 8). Numbers with a letter in common within a row are not significantly different (*p* = 0.05).

Faecal Parameter	Diet				*p*	F
	18.6% Casein	37.2% Casein	Meat	Pea		
Dry matter (g/100 g diet)	5.09 ± 0.40 a	5.23 ± 0.33 a	5.29 ± 0.27 a	11.6 ± 0.99 b	<0.001	264
Water-holding capacity (mL/g)	3.06 ± 0.18 b	3.47 ± 0.20 c	2.73 ± 0.14 a	5.11 ± 0.63 d	<0.001	99.5
Hydrated faecal mass (HFM) (g/100 g diet)	20.7 ± 1.68 ab	23.4 ± 2.09 b	19.7 ± 0.85 a	71.4 ± 13. 3 c	<0.001	247
Mean change in HFM from 18.6% casein (g)	0	2.7	−1	50.7		

**Table 3:** Mass of contents (g) in three regions of the hindgut of rats fed diets containing four protein sources (*n* = 8, means ± SD). Numbers with a letter in common are not significantly different (*p* = 0.05).

Gut Region	Diet				*p*	F
	18.6% Casein	37.2% Casein	Meat	Pea		
Caecum	2.59 ± 0.79 a	2.84 ± 0.56 a	2.77 ± 0.32 a	5.51 ± 0.74 b	<0.001	21.3
Proximal colon	0.42 ± 0.25 ab	0.17 ± 0.26 a	0.10 ± 0.19 a	0.73 ± 0.49 b	0.002	6.49
Distal colon	0.73 ± 0.33 b	0.88 ± 0.35 b	0.20 ± 0.16 a	1.44 ± 0.29 c	<0.001	24.92

**Table 4:** Short-chain fatty acids in caecal contents (µmol/g fresh weight) from rats fed diets containing 18.6% casein, 37.2% casein, 18.6% meat protein, and 18.6% protein from pea cotyledon, ranked in order of caecal concentration in pea-fed rats (means ± SD). ANOVA on log-transformed data, with 4 and 23 degrees of freedom.

Organic Acid		Diet				F	*p*
		18.6% Casein (*n* = 5)	37.2% Casein (*n* = 7)	Meat (*n* = 7)	Pea (*n* = 8)		
Acetate	Mean	36.58	46.00	58.25	67.93		
	SD	10.8	12.75	12.01	16.08	7.07	0.002
	Tukey	a	ab	bc	c		
Butyrate	Mean	6.11	8.21	9.52	26.71		
	SD	1.74	1.83	2.10	6.18	51.04	<0.001
	Tukey	a	ab	b	c		
Propionate	Mean	12.66	13.89	18.02	22.04		
	SD	2.87	3.97	3.54	6.77	4.95	0.009
	Tukey	a	a	ab	b		
Lactate	Mean	6.15	6.05	2.09	11.69		
	SD	10.73	10.07	1.15	4.81	5.40	0.006
	Tukey	a	a	a	b		
Succinate	Mean	2.87	7.06	3.00	4.21		
	SD	1.27	7.474	2.73	4.04	0.38	0.770
	Tukey						
Heptanoate	Mean	1.82	1.87	1.62	1.82		
	SD	0.07	0.18	0.63	0.08	0.87	0.472
	Tukey						
Isobutyrate	Mean	1.96	2.38	2.43	1.32		
	SD	0.08	0.28	0.48	0.21	25.53	<0.001
	Tukey	b	b	b	a		
Formate	Mean	1.40	2.06	2.00	1.20		
	SD	0.61	1.56	0.93	0.41	1.67	0.201
	Tukey						
Isovalerate	Mean	0.48	0.54	0.85	0.19		
	SD	0.13	0.20	0.35	0.04	21.69	<0.001
	Tukey	b	b	b	a		
Valerate	Mean	1.20	1.69	2.04	0.78		
	SD	0.21	0.70	0.59	0.08	4.89	0.009
	Tukey	ab	ab	b	a		
Hexanoate	Mean	0.20	0.20	0.20	0.59		
	SD	0	0	0	0.5	5.74	0.004
	Tukey	a	a	a	b		

Means with a letter in common do not differ significantly (*p* < 0.05).

## Data Availability

The original contributions presented in this study are included in the article/Supplementary Material. Further inquiries can be directed to the corresponding author.

## Supplementary Materials

The following supporting information can be downloaded at: https://www.mdpi.com/article/10.3390/nu18203347/s1, Table S1. ANOVA of caecal protein putrefaction products. Table S2. Indole concentration (µg/mL) by time of secondary fermentation ANOVA (LSD = 1.18). Table S3. *p*-cresol concentration (µg/mL) by time of secondary fermentation ANOVA (LSD = 0.99). Table S4. Phenol concentration (µg/mL) by time of secondary fermentation ANOVA (LSD = 3.34). Table S5. Determination of the survival of pea non-digested polysaccharide (NDP) after gastro-intestinal transit and the proportion of faecal NDP that originated from pea. Figure S1. Feed intakes over 4-day balance period with 25 g diet allocation per day (Means ± SD). Figure S2. Group (*n* = 8) average rat weights over 9-days ingestion of the four treatments (Mean ± SD). The trend line is for the mean of all rats at each measurement point. The meat and pea treatments each contained 18.6% protein. Figure S3. Dry weight of faeces over 4-day balance period during which all rats were allocated 25 g per day and all groups consumed an average of 80 g of diet per day (Means ± SD). Figure S4. Faecal water-holding capacity measured as water absorbed during 24 h hydration in excess water by dry faecal pellets from rats fed four different diets (Means ± SD). Figure S5. Hydrated faecal mass calculated as the dry matter output adjusted to a 100 g feed intake plus the water bound by the fully hydrated faecal mass (Faecal dry weight per 100 g diet ingested + (faecal dry weight per 100 g diet ingested × water-holding capacity per gram) (Mean ± SD). Figure S6. Pattern of differences in putrefaction product concentrations throughout the hind gut—caecum, proximal colon, distal colon. Casein, meat and pea diets contained 18.6% protein, Casein × 2 contained 37.2% protein. Figure S7. Origin of faecal non-digested polysaccharide (NDP) as a percentage of faecal dry matter. Based on duplicate analyses of the bulked samples used for secondary fermentation.
