# Supplementary material for: Dietary Fibre in Pea Cotyledon Used as a Protein Source Provides Multidimensional Protection Against Colonic Protein Putrefaction
Source: Nutrients. 2026 Oct 13;18(20):3347. doi: 10.3390/nu18203347 (PMC13708442; doi:10.3390/nu18203347)
Supplement: Supplementary file 1 [file nutrients-18-03347-s001.zip › nutrients-4511442-supplementary.pdf]

SUPPLEMENTARY

Table S1. ANOVA of caecal protein putrefaction products.

ANOVA	p-cresol		indole		phenol		ammonia	
	F	p	F	p	F	p	F	p
Treatment (3 and 28 df)	4.3	0.014	8	<.001	5	0.007	18	<.001
Means		Tukey letters		Tukey letters		Tukey letters		Tukey letters
18.6% casein	8.2	ab	5.9	b	17	b	26.68	b
37.2% casein	10.1	ab	7.2	b	19.3	b	30.16	b
Meat	13.9	b	9.4	b	16.3	ab	27.19	b
Pea	4.8	a	0.7	a	5.3	a	14.29	a

Tables S2–S4: Release of putrefaction products during secondary digestion of rat faeces with human faecal inoculum, with (+P) and without added protein. CaseinL = 18.6% casein, CaseinH = 37.2% casein. F = faeces.

Table S2. Indole concentration (μg/mL) by time of secondary fermentation ANOVA (LSD = 1.18).

		Time of fermentation									
		0		8		16		24		32	
	Group	Mean	SD	Mean	SD	Mean	SD	Mean	SD	Mean	SD
1	CaseinLF	0.26	0.08	8.71	0.36	7.34	0.97	8.72	0.12	7.54	0.22
2	CaseinHF	0.10	0.09	7.45	2.74	7.80	0.18	9.19	0.17	8.83	0.90
3	BeefF	0.18	0.05	9.13	0.28	6.76	0.39	8.18	1.25	6.58	2.01
4	PeaF	0.00	0.00	4.15	0.64	3.19	0.29	2.97	0.48	2.14	1.25
5	Water	0.00	0.00	8.08	0.47	7.22	0.37	6.89	0.74	6.21	0.92
6	Inulin	0.00	0.00	1.17	0.05	0.40	0.13	0.23	0.12	0.06	0.10
7	CaseinLF+P	0.17	0.02	9.85	0.14	11.08	0.77	12.90	0.82	12.54	1.19
8	CaseinHF+P	0.19	0.05	10.16	0.58	12.76	0.46	13.91	0.48	14.15	0.67
9	BeefF+P	0.25	0.02	10.39	0.30	11.50	0.79	12.74	0.57	12.97	0.85
10	PeaF+P	0.00	0.00	2.16	0.46	2.52	0.33	4.37	0.44	5.30	0.56
11	Water+P	0.05	0.08	8.26	0.32	9.71	0.05	12.23	0.12	13.57	1.04
12	Inulin+P	0.00	0.00	0.96	0.01	0.48	0.11	0.34	0.14	0.11	0.19
	p	1		<.001		<.001		<.001		<.001	

Analysis of variance

Variate	: p_cresol 0	p_cresol 8	p_cresol 16	p_cresol 24	p_cresol 32
Source of variation	d.f.	s.s.	m.s.	v.r.	F pr.
Subject stratum					
C2	11	2019.2938	183.5722	324.91	<.001
Residual	24	13.56	0.565	1.31	
Subject.Time stratum					
d.f. correction factor 0.6487					
Time	4	1462.1471	365.5368	847.51	<.001
Time.C2	44	628.0476	14.2738	33.09	<.001

Accumulated analysis of variance

Change	d.f.	s.s.	m.s.	v.r.	F pr.
TIME	4	1432.8423	358.2106	830.52	<.001
treat0	11	0.3551	0.0323	0.07	1
treat8	11	423.0806	38.4619	89.18	<.001
treat16	11	593.8553	53.9868	125.17	<.001
treat24	11	777.5602	70.6873	163.89	<.001
treat32	11	852.513	77.5012	179.69	<.001
animal	24	13.4787	0.5616	1.3	0.185
Residual	95	40.9741	0.4313		

Table S3. p-cresol concentration (µg/mL) by time of secondary fermentation ANOVA (LSD = 0.99).

		Time of fermentation (h)									
		0		8		16		24		32	
	Group	Mean	SD	Mean	SD	Mean	SD	Mean	SD	Mean	SD
1	CaseinLF	0.57	0.15	0.67	0.03	0.76	0.10	1.18	0.15	1.12	0.04
2	CaseinHF	0.00	0.00	0.49	0.42	0.90	0.12	1.43	0.40	1.78	1.01
3	BeefF	0.59	0.14	1.00	0.11	1.22	0.19	2.24	0.31	2.44	0.32
4	PeaF	0.50	0.02	0.63	0.10	0.88	0.35	1.37	0.39	1.33	0.59
5	Water	0.00	0.00	0.45	0.02	0.73	0.15	0.69	0.08	0.74	0.19
6	Inulin	0.00	0.00	0.14	0.24	0.00	0.00	0.61	0.11	0.42	0.03
7	CaseinLF+P	0.55	0.04	0.85	0.19	2.28	0.10	7.20	0.24	14.10	1.17
8	CaseinHF+P	0.29	0.25	0.76	0.09	2.27	0.13	7.35	0.89	15.70	1.79
9	BeefF+P	0.66	0.12	1.50	0.07	2.45	0.24	7.87	0.47	17.58	1.20
10	PeaF+P	0.16	0.28	0.46	0.40	1.28	0.25	4.73	0.65	12.34	1.14
11	Water+P	0.16	0.28	0.56	0.11	2.13	0.55	7.64	1.36	17.79	1.31
12	Inulin+P	0.00	0.00	0.35	0.31	0.13	0.23	0.34	0.30	0.56	0.17
	p	0.375		0.049		<.001		<.001		<.001	

Analysis of variance

Variate	: p_cresol 0	p_cresol 8	p_cresol 16	p_cresol 24	p_cresol 32
Source of variation	d.f.	s.s.	m.s.	v.r.	F pr.
Subject stratum					
C2	11	906.4953	82.4087	148.34	<.001
Residual	24	13.3331	0.5555	2.85	
Subject.Time stratum					
d.f. correction factor 0.3592					
Time	4	1174.062	293.5155	1504.51	<.001
Time.C2	44	1312.713	29.8344	152.93	<.001
Residual	96	18.7287	0.1951		
Total	179	3425.332			

Accumulated analysis of variance

Change	d.f.	s.s.	m.s.	v.r.	F pr.
TIME	4	1174.062	293.5155	1504.51	<.001
treat0	11	2.3465	0.2133	1.09	0.375
treat8	11	4.065	0.3695	1.89	0.049
treat16	11	23.659	2.1508	11.02	<.001
treat24	11	324.7983	29.5271	151.35	<.001
treat32	11	1864.339	169.4854	868.75	<.001
animal	24	13.3331	0.5555	2.85	<.001
Residual	96	18.7287	0.1951		

Table S4. Phenol concentration (μg/mL) by time of secondary fermentation ANOVA (LSD = 3.34).

		Time of fermentation (h)									
		0		8		16		24		32	
	Group	Mean	SD	Mean	SD	Mean	SD	Mean	SD	Mean	SD
1	CaseinLF	0.00	0.00	0.21	0.37	1.36	0.24	2.94	0.35	3.84	0.76
2	CaseinHF	0.00	0.00	0.00	0.00	4.09	4.10	6.50	4.01	8.04	3.32
3	BeefF	0.00	0.00	1.04	0.11	1.74	0.33	3.30	1.69	4.31	2.36
4	PeaF	0.00	0.00	0.00	0.00	1.43	0.48	1.49	1.29	1.66	1.45
5	Water	0.00	0.00	0.00	0.00	1.11	0.29	1.32	1.14	1.49	1.38
6	Inulin	0.00	0.00	0.00	0.00	0.00	0.00	0.00	0.00	0.00	0.00
7	CaseinLF+P	0.00	0.00	1.16	0.27	8.22	1.76	10.84	2.26	13.80	2.84
8	CaseinHF+P	0.00	0.00	1.06	0.05	12.97	2.73	15.54	2.55	18.73	3.15
9	BeefF+P	0.00	0.00	1.40	0.24	10.75	2.35	14.12	2.59	18.17	3.65
10	PeaF+P	0.00	0.00	0.00	0.00	9.20	4.50	13.81	4.22	17.31	3.08
11	Water+P	0.00	0.00	0.22	0.37	6.25	0.89	8.89	1.07	14.09	1.90
12	Inulin+P	0.00	0.00	0.00	0.00	0.00	0.00	0.00	0.00	0.00	0.00
	p	1		0.838		<.001		<.001		<.001	

Analysis of variance

Variate	: p_cresol 0	p_cresol 8	p_cresol 16	p_cresol 24	p_cresol 32
Source of variation	d.f.	s.s.	m.s.	v.r.	F pr.
Subject stratum					
C2	11	2173.519	197.593	23.03	<.001
Residual	24	205.881	8.578	5.36	
Subject.Time stratum					
d.f. correction factor 0.3296					
Time	4	2007.1	501.775	313.32	<.001
Time.C2	44	1497.221	34.028	21.25	<.001
Residual	96	153.743	1.601		
Total	179	6037.463			

Accumulated analysis of variance

Change	d.f.	s.s.	m.s.	v.r.	F pr.
TIME	4	2007.1	501.775	313.32	<.001

treat0	11	0	0	0	1
treat8	11	10.284	0.935	0.58	0.838
treat16	11	684.195	62.2	38.84	<.001
treat24	11	1131.201	102.836	64.21	<.001
treat32	11	1845.059	167.733	104.74	<.001
animal	24	205.881	8.578	5.36	<.001
Residual	96	153.743	1.601		

Table S5. Determination of the survival of pea non-digested polysaccharide (NDP) after gastro-intestinal transit and the proportion of faecal NDP that originated from pea.

	**Diet**			
	18.6% casein	37.2% casein	Meat	Pea
NSP intake/100 g diet (g):				
Basal	2.93	2.93	2.93	2.93
Pea	0	0	0	15.93
Total	2.93	2.93	2.93	19.73
Faceal dry weight /100 g diet (g)	5.09	5.23	5.29	11.61
Faecal NDP, g /g faeces	0.358	0.371	0.369	0.355
Faecal NDP/100 g diet	1.82	1.94	1.95	4.12
Pea-derived NDP/100 g diet	-0.08	0.04	0.05	2.22
% faecal dry matter as NDP	35.8	37.1	36.9	35.5
% faecal NDP from Pea	0.0	0.0	0.0	53.9
% faecal dry matter as pea NDP	0	0	0	19.1
% faecal dry matter non-pea NDP	35.8	37.1	36.9	16.4
% apparent NDP survival	62.2	66.2	66.6	20.9

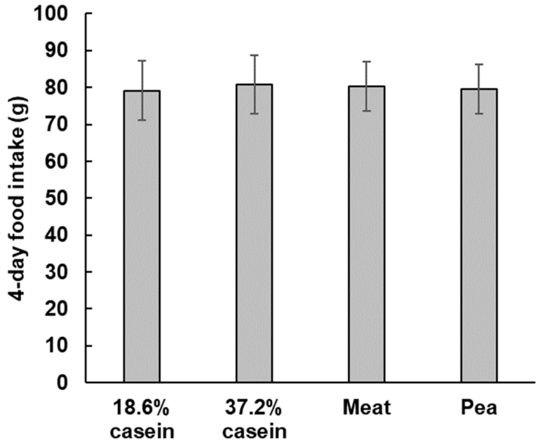

Figure S1. Feed intakes over 4-day balance period with 25 g diet allocation per day (Means ± SD).

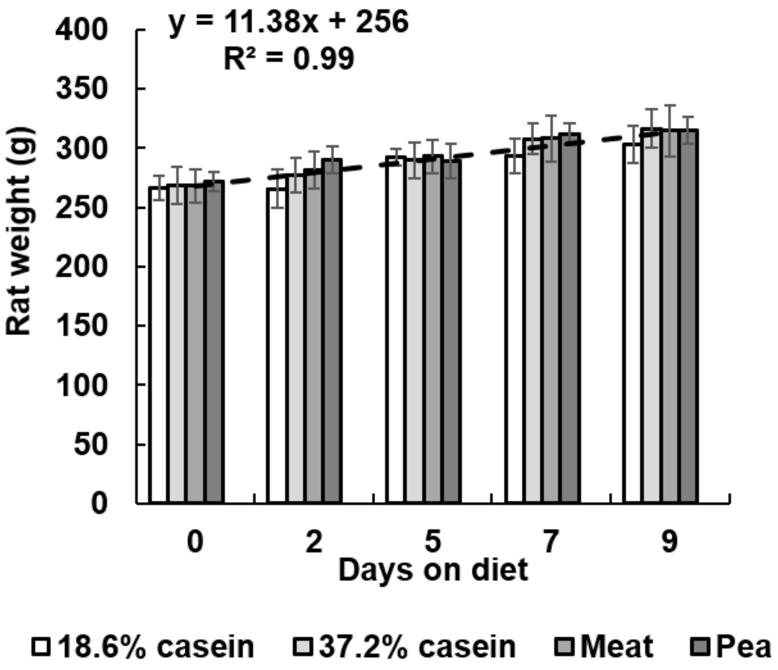

Figure S2. Group (n = 8) average rat weights over 9-days ingestion of the four treatments (Mean ± SD). The trend line is for the mean of all rats at each measurement point. The meat and pea treatments each contained 18.6% protein.

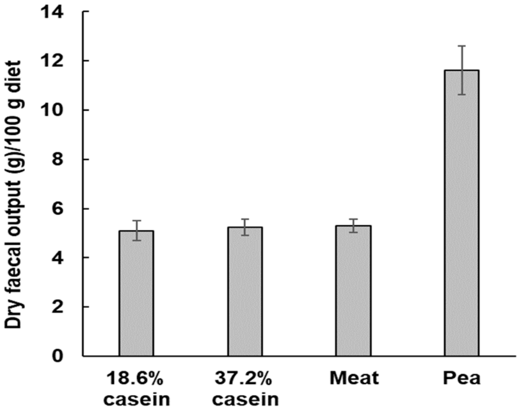

Figure S3. Dry weight of faeces over 4-day balance period during which all rats were allocated 25 g per day and all groups consumed an average of 80 g of diet per day (Means ± SD).

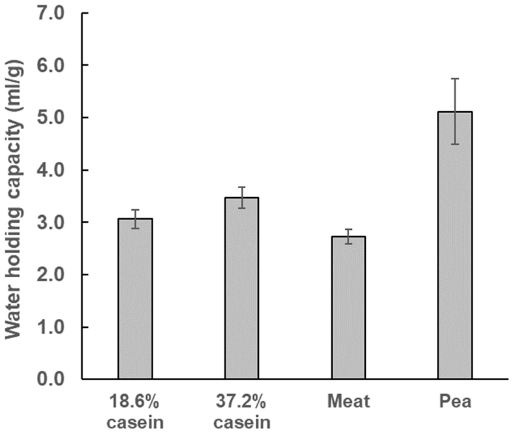

Figure S4. Faecal water-holding capacity measured as water absorbed during 24 h hydration in excess water by dry faecal pellets from rats fed four different diets (Means ± SD).

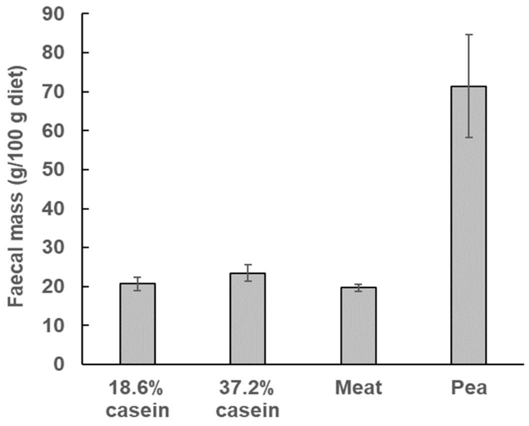

Figure S5. Hydrated faecal mass calculated as the dry matter output adjusted to a 100 g feed intake plus the water bound by the fully hydrated faecal mass (Faecal dry weight per 100 g diet ingested + (faecal dry weight per 100 g diet ingested × water-holding capacity per gram) (Mean ± SD).

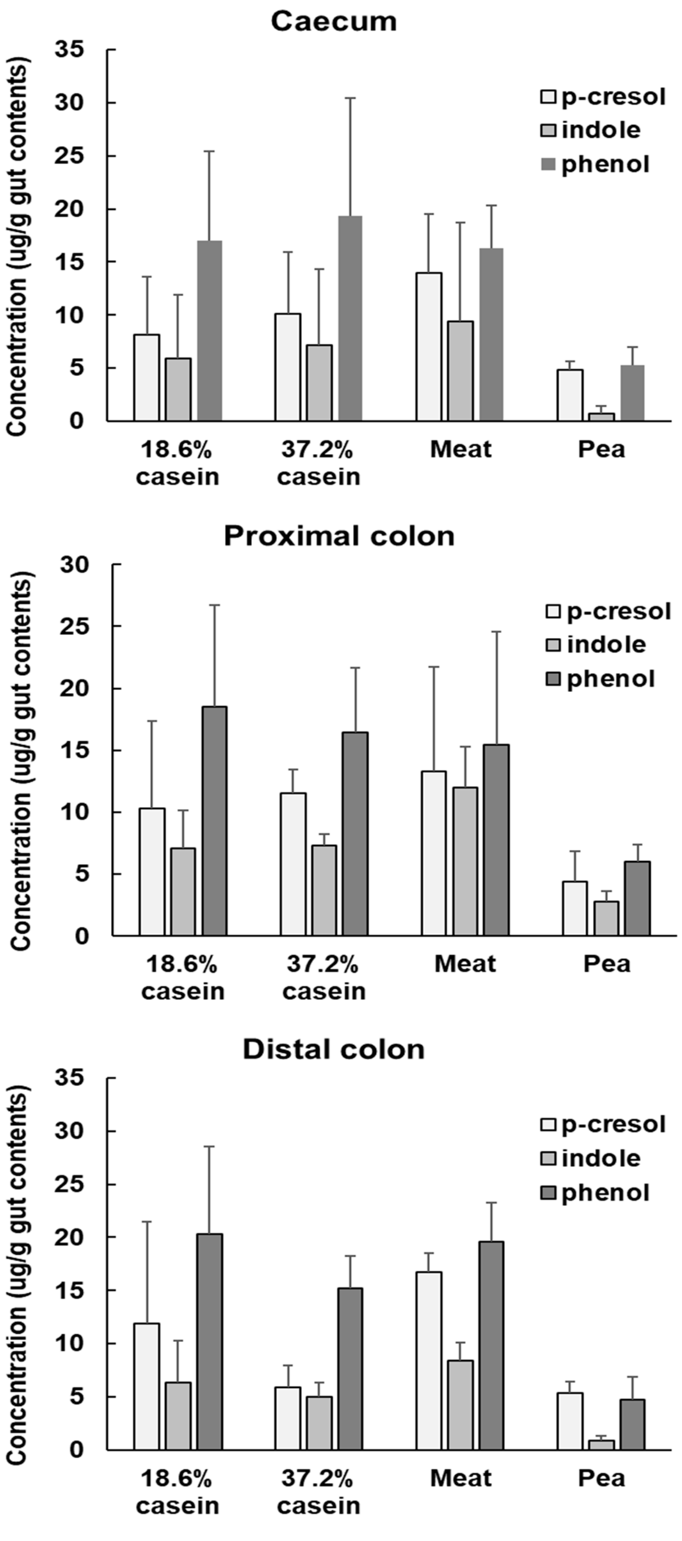

Figure S6. Pattern of differences in putrefaction product concentrations throughout the hind gut—caecum, proximal colon, distal colon. Casein, meat and pea diets contained 18.6% protein, Casein × 2 contained 37.2% protein.

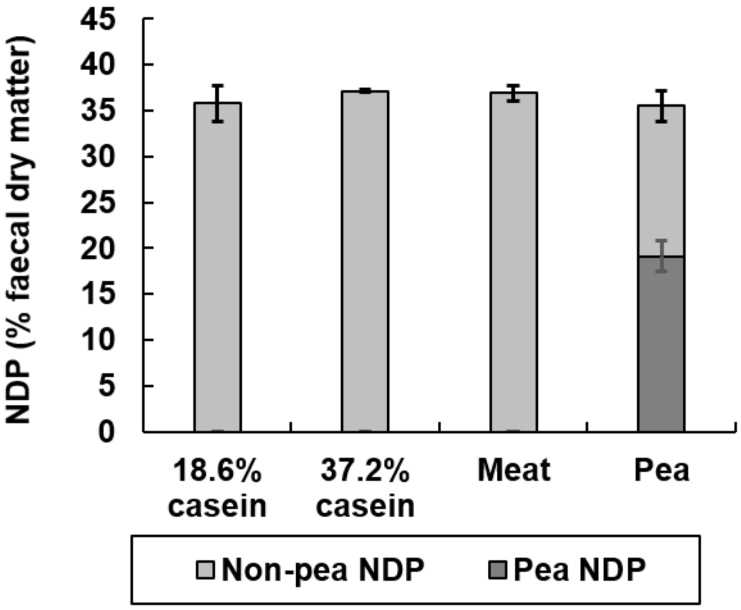

Figure S7. Origin of faecal non-digested polysaccharide (NDP) as a percentage of faecal dry matter. Based on duplicate analyses of the bulked samples used for secondary fermentation.
